# Joint association of alcohol consumption and adiposity with alcohol- and obesity-related cancer in a population sample of 399,575 UK adults

**DOI:** 10.1017/S0007114522003464

**Published:** 2023-08-14

**Authors:** Elif Inan-Eroglu, Bo-Huei Huang, Peter Sarich, Natasha Nassar, Emmanuel Stamatakis

**Affiliations:** 1 Charles Perkins Centre, School of Health Sciences, Faculty of Medicine and Health, The University of Sydney, Sydney, NSW, Australia; 2 Department of Molecular Epidemiology, German Institute of Human Nutrition Potsdam-Rehbruecke, Germany; 3 The Daffodil Centre, The University of Sydney, A Joint Venture with Cancer Council NSW, Sydney, NSW, Australia; 4 Charles Perkins Centre, Faculty of Medicine and Health, The University of Sydney, Camperdown, Sydney, NSW, Australia

**Keywords:** Cancer, Alcohol consumption, Adiposity, Obesity

## Abstract

Obesity and alcohol consumption are both important modifiable risk factors for cancer. We examined the joint association of adiposity and alcohol consumption with alcohol- and obesity-related cancer incidence. This prospective cohort study included cancer-free UK Biobank participants aged 40–69 years. Alcohol consumption was categorised based on current UK guidelines into four groups. We defined three markers of adiposity: body fat percentage (BF %), waist circumference and BMI and categorised each into three groups. We derived a joint alcohol consumption and adiposity marker variable with twelve mutually exclusive categories. Among 399 575 participants, 17 617 developed alcohol-related cancer and 20 214 developed obesity-related cancer over an average follow-up of 11·8 (SD 0·9) years. We found relatively weak evidence of independent associations of alcohol consumption with cancer outcomes. However, the joint association analyses showed that across all adiposity markers, above guideline drinkers who were in the top two adiposity groups had elevated cancer incidence risk (e.g. HR for alcohol-related cancer was 1·53 (95 % CI (1·24, 1·90)) for within guideline drinkers and 1·61 (95 % CI (1·30, 2·00)) for above guideline drinkers among participants who were in the top tertile BF %. Regardless of alcohol consumption status, the risk of obesity-related cancer increased with higher adiposity in a dose–response manner within alcohol consumption categories. Our study provides guidance for public health priorities aimed at lowering population cancer risk via two key modifiable risk factors.

Cancer constitutes the second leading cause of death worldwide, accounting for nearly 10 million deaths in 2020^(1)^. There is overwhelming evidence that lifestyle risk factors impact cancer risk and that population-wide changes can significantly reduce cancer burden^(2)^. Owing to the impact of modifiable risk factors, especially for the most prevalent cancers, more than half of cancers occurring today may be preventable^(3)^. Among the key modifiable risk factors for cancer are alcohol consumption and adiposity^(4)^.

Alcohol is classified as a Group 1 carcinogen (highest level) by the International Agency for Research on Cancer (IARC)^(5)^ and has been strongly linked to many common cancer incidence and mortality^(6–8)^. Globally, 4·1 % (741 300 cases) of all new cancer cases in 2020 were attributable to alcohol consumption^(8)^. Although current evidence suggests that the safest level of alcohol consumption is total avoidance^(9)^, alcohol consumption has been rising in many countries^(10,11)^. Adiposity is also another major contributor to cancer risk with a dramatic rise in prevalence of obesity worldwide^(12,13)^. A review on human, animal and mechanistic studies conducted by IARC showed that overweight or obesity increases the risk of at least thirteen types of cancer that accounted for 42 % of all new cancer diagnoses worldwide^(14)^.

Although alcohol consumption and adiposity have been separately investigated as cancer risk factors^(15–18)^, very few studies have investigated the joint association between alcohol consumption and adiposity with cancer risk^(19–21)^. Loomba *et al*.^(20)^ showed that body mass index (BMI) defined obesity and alcohol have synergistic effects of increasing the risk of hepatocellular carcinoma. In a study of colorectal cancer risk^(21)^, alcohol was associated with a higher risk among obese participants (BMI ≥ 30 kg/m^2^). Most of these studies focused on a single cancer site^(19,20)^ or were retrospective case–control studies^(21)^. Additionally, the majority of studies focused on BMI^(19–21)^. No study has examined the association of alcohol and excess adiposity on the full range of cancers that are known to be influenced by alcohol intake and obesity.

The aim of this study was to investigate the joint association of excess adiposity and alcohol on alcohol-related, obesity-related and total cancer incidence in a large population sample of UK adults with no cancer diagnosis at baseline.

## Methods

### Study population

This research was conducted using the UK Biobank, a population-based prospective cohort study. About 9·2 million invitations were mailed to recruit participants aged 40–69 years between 2006 and 2010 from twenty-two centres across the UK^(22)^. This study was conducted according to the guidelines laid down in the Declaration of Helsinki, and all procedures involving human subjects were approved by the National Health Service (NHS), National Research Ethics Service (Ref 11/NW/0382). Written informed consent was obtained from all subjects.

We excluded participants with missing data on any exposures or covariates. We also excluded participants with a baseline diagnosis of cancer (International Classification of Diseases, Tenth Revision (ICD-10), C00·0 to C97) and underweight participants (BMI < 18·5 kg/m^2^) from all analyses to reduce the possibility of confounding by poor health status-related weight loss (online Supplementary Table 1)^(23)^.

### Alcohol consumption and adiposity

Alcohol consumption was self-reported as the number of UK units per week (average weekly intake of red wine + champagne and white wine + beer and cider + spirits + fortified wine + and other). In the UK, one unit is equal to 8 g of ethanol. Participants were grouped based on the current UK alcohol guidelines^(24)^: (1) never drinkers; (2) previous drinkers; (3) within guideline drinkers (≤ 14 UK units of alcohol/week); and (4) above guideline drinkers (> 14 UK units)^(25)^. Previous drinkers included participants who stopped drinking due to illness or ill health (23·5 %), doctor’s advice (5·2 %), health precaution (19·8 %), financial or other reasons (1·8 %) and other reasons (49·7 %). Never drinkers included participants who never drank alcohol in their lifetime.

Adiposity was defined using measures of body fat percentage (BF %), waist circumference (WC) and BMI. BF % was measured by bioimpedance using the Tanita BC-418MA device (Tanita). We categorised BF % based on the sex-specific tertiles of BF %. WC at the level of the umbilicus was measured by a trained professional using a Wessex non-stretchable sprung tape, with the participant in the resting-standing position. WC was categorised as: (1) normal (< 80 cm for women, < 94 cm for men); (2) increased risk of metabolic complications (80–88 cm for women, 94–102 cm for men); and (3) substantially increased risk of metabolic complications (> 88 cm for women, > 102 cm for men)^(26)^. Trained staff measured body weight and height, and BMI was calculated as weight (in kilograms) divided by height squared (in metres). For the analyses, individuals were categorised into three groups: (1) normal weight (18·5–24·9 kg/m^2^); (2) overweight (25·0–29·9 kg/m^2^); and (3) obese (≥ 30·0 kg/m^2^).

For the joint alcohol consumption and adiposity marker analyses, we combined the four alcohol consumption and three adiposity marker categories to create a variable with twelve mutually exclusive categories.

### Outcomes

We identified alcohol- and obesity-related cancers from hospital admissions and cancer registry data. These were coded according to the International Classification of Diseases 9th (ICD-9)^(27)^ and 10th Revision (ICD-10)^(28)^ diagnosis codes (listed in the Appendix). Hospital admission data were censored in March 2020 for England and Scotland and February 2018 for Wales. Cancer registry data were censored in July 2019 for England and Scotland and October 2015 for Wales. Participants were followed up from the date of attendance at the recruitment centre to the date of cancer incidence or date of censorship, whichever came first. Cancer registry data were obtained from the National Health Service (NHS) Information Centre (England and Wales) and the NHS Central Register Scotland (Scotland) and were linked to survey data. In line with a previous study^(29)^, we classified alcohol-related cancers using two definitions, depending on the strength of evidence for their causal relationship with alcohol in existing literature and IARC^(6,30–33)^:

- A narrow definition included oral cavity, throat, larynx, oesophagus, liver, colorectal, stomach and female breast cancer.
- A broader definition included all above plus pancreas and lung cancers, which have less consistent evidence of an association with alcohol.

Throughout the manuscript, alcohol-related cancer indicates the narrow definition, unless otherwise stated. We used IARC’s definition for obesity-related cancers^(14)^, including oesophagus, gastric cardia, colorectal, liver, gallbladder, pancreas, female breast, corpus uteri, ovary, kidney, thyroid and multiple myeloma. We also combined alcohol-related and obesity-related cancer into one outcome (fifteen sites in total: oral cavity, throat, larynx, oesophagus, liver, colorectal, stomach, female breast, gallbladder, pancreas, corpus uteri, ovary, kidney, thyroid and multiple myeloma) which we referred as ‘combined cancers’ in the text. In addition, we present results for cancers that are both obesity- and alcohol-related (five sites: oesophagus, liver, colorectal, gastric cardia and breast cancers). We refer to this outcome as ‘overlapping cancers’ throughout the text.

### Covariates

We adjusted analyses using information collected from baseline surveys, including age (years), sex, dietary pattern (calculated using information on consumption of fruit, vegetables, fish, unprocessed red meat and processed meat)^(34–36)^, education (college/university degree; higher school certificate or equivalent; school certificate or equivalent; secondary education certificate or equivalent; vocational qualification, higher diploma/certificate, or equivalent; other professional qualifications), physical activity (International Physical Activity Questionnaire (IPAQ)^(37)^ derived MET-hours per week), smoking (never, previous and current smokers), sleep duration (h/d), socio-economic status (Townsend Deprivation Index^(38)^) and chronic illness (the presence/absence of CVD (ICD-10 codes I00 to I99) or type 2 diabetes (E11·0 to E11·9 and E12) using self-reported doctor diagnosis and hospital admission records).

### Statistical analyses

We used Cox proportional hazard models to estimate associations of exposures and outcomes. We examined the proportional hazards assumption using Kaplan–Meier survival plots^(39)^ and Schoenfeld residuals, and we noted no apparent violations (*P* > 0·05 for the association of alcohol consumption and adiposity markers with cancer risk). Firstly, we examined the independent association of alcohol consumption and adiposity markers with all study outcomes, using never drinkers and the bottom group of each adiposity marker as referent groups, respectively. We then examined the joint association of adiposity and alcohol consumption with the incidence of alcohol-related, obesity-related and total cancers using the ‘optimal’ group of the joint variable as the referent group (never drinker and lowest adiposity marker group of each adiposity marker). The first model was adjusted for sex and baseline age. The second model was further adjusted for smoking, diet, education, sleep, socio-economic status and physical activity. The fully adjusted model also included chronic illness (CVD and type 2 diabetes). We also mutually adjusted for BMI and alcohol consumption in the independent effects of alcohol and adiposity analyses. We tested for statistical interaction by entering an alcohol consumption*adiposity marker (BF %, WC or BMI) term in the first model. In a sensitivity analysis, we excluded events in the first 3 years of follow-up from analyses of the joint association between alcohol consumption and adiposity markers with obesity- and alcohol-related cancer incidence to minimise the possibility of reverse causation bias. We also examined the joint association between alcohol consumption and adiposity markers with incidence for each alcohol-related cancer site. As smoking and alcohol consumption can synergistically increase the risk of cancer^(40)^, we tested for a three-way statistical interaction for alcohol consumption*adiposity marker (BF %, WC or BMI)*smoking status in the first model. We then examined the joint association between smoking status and alcohol consumption with alcohol-related cancer incidence. For this analysis, we grouped smoking status as never, previous, and current smokers and derived a joint alcohol consumption and smoking status variable with twelve mutually exclusive categories. We used the ‘optimal’ group of the joint variable as the referent group (never drinker and never smoker group). All analyses were performed using R statistical software. We reported this study as per the Strengthening the Reporting of Observational Studies in Epidemiology (STROBE) guideline (Supplemental STROBE Checklist).

## Results

Table 1 shows the baseline characteristics of the sample (*n* 399 575). The mean age was 56·2 (sd: 8·1) years, and 55 % were females. Participants had an average BF % of 31·6 %, a WC of 90·4 cm and a BMI of 27·5 kg/m^2^. Over an average follow-up time of 11·8 (0·9) years a total of 61 898 incident cancer events were identified, including 22 211/17 616 alcohol-related (broad/narrow definition), and 20 214 obesity-related cancer events. We found an interaction effect between alcohol consumption and WC (*P* = 0·015) and BMI (*P* = 0·002) for broad definition of alcohol-related cancer and total cancer (*P* < 0·001 for both adiposity markers) (online Supplementary Table 1).

**Table 1.** Baseline characteristics of study sample by alcohol consumption category (*n* 399 575)

	Overall		Never drinker		Previous drinker		Within guidelines drinker		Above guidelines drinker	
	Mean	sd	Mean	sd	Mean	sd	Mean	sd	Mean	sd
Total *n*	399 575		17 061		13 648		224 171		144 695	
Weekly alcohol unit	13·7	16·6	–		–		5·1	4·6	30·0	17·6
Age	56·2	8·1	56·7	8·6	56·9	7·9	56·2	8·1	56·1	7·9
	*n*	%	*n*	%	*n*	%	*n*	%	*n*	%
Male	179 944	45·0	4804	28·2	6031	44·2	76 234	34·0	92 875	64·2
	Mean	sd	Mean	sd	Mean	sd	Mean	sd	Mean	sd
Follow-up*	11·8	0·9	11·7	0·9	11·8	0·9	11·8	0·9	11·8	0·9
Sleep	7·2	1·1	7·1	1·3	7·1	1·4	7·2	1·1	7·2	1·0
Dietary pattern score (DPS)†	1·7	0·5	1·7	0·5	1·7	0·5	1·7	0·5	1·6	0·5
	*n*	%	*n*	%	*n*	%	*n*	%	*n*	%
Chronic disease‡	169 133	42·3	8450	49·5	7178	52·6	90 256	40·3	63 249	43·7
Socio-economic status	-1·4	3·1	-0·2	3·5	-0·1	3·5	-1·4	3·0	-1·5	2·9
Smoking status										
Never	221 457	55·4	14 016	82·2	6162	45·1	138 492	61·8	62 787	43·4
Current	137 320	34·4	1999	11·7	5403	39·6	67 054	29·9	62 864	43·4
Previous	40 798	10·2	1046	6·1	2083	15·3	18 625	8·3	19 044	13·2
	Mean	SD	Mean	SD	Mean	SD	Mean	SD	Mean	SD
Physical activity§	251·9	226·8	218·8	225·5	236·0	233·9	246·7	225·9	265·2	226·9
Body fat percentage (BF %)‖	31·6	8·5	34·7	8·8	32·8	9·2	32·8	8·6	29·3	7·7
BMI¶	27·5	4·8	28·3	5·5	28·5	5·7	27·4	5·0	27·4	4·2
Waist circumference (WC) (cm)**	90·4	13·4	90·4	14·1	92·8	14·9	88·9	13·5	92·4	12·7
	*n*	%	*n*	%	*n*	%	*n*	%	*n*	%
Narrow definition of alcohol-related cancer‡‡	17 616	4·4	742	4·3	691	5·1	10 172	4·5	6111	4·2
Broad definition of alcohol-related cancer††	22 211	5·6	922	5·4	966	7·1	12 362	5·5	7961	5·5
Obesity-related cancer§§	20 124	5·0	962	5·6	755	5·5	11 832	5·3	6575	4·5
Total cancer‖‖	61 898	15·5	2229	13·1	2215	16·2	33 362	14·9	24 092	16·7

* Follow-up years until cancer incident or end of the study, whichever came first.

† DPS was determined by higher consumption of fruit, vegetables, and fish and lower consumption of processed meats and red meats (Rutten-Jacobs *et al*., 2018).

‡ Major CVD (ICD-10 codes I00 to I99), type 2 diabetes (ICD-10 codes E11·0 to E11·9 and E12) and dyslipidaemia (ICD-10 codes E78·0–E78·6) diagnosed by a doctor and hospital admission records.

§ Physical activity (PA) patterns were based on the frequency and duration of PA in the 4 weeks prior to the survey. PA was quantified in Metabolic Equivalent Task (MET)-h/week, computed by multiplying the activity by the MET value and then summing the number of MET-hours spent performing each activity per week.

‖ BF % was measured by bioimpedance using the Tanita BC-418MA device (Tanita).

¶ BMI = Weight (kg)/height (m^2^).

** Waist circumference were measured by using flexible plastic tape with the participant in the resting-standing position by a trained professional.

†† Alcohol-related cancer according to the broad definition included oral cavity, throat, larynx, oesophagus, liver, colorectal, stomach, female breast, pancreas and lung cancer (IARC, 2014).

‡‡ Alcohol-related cancer according to the narrow definition included oral cavity, throat, larynx, oesophagus, liver, colorectal, stomach and female breast (IARC, 2014).

§§ Obesity-related cancer included meningioma, multiple myeloma, adenocarcinoma of the oesophagus, and cancers of the thyroid, postmenopausal breast, gallbladder, stomach, liver, pancreas, kidney, ovaries, uterus, colon and rectum (colorectal) (Lauby-Secretan *et al*., 2016).

‖‖ The definition of total cancer excludes *in situ*, benign, uncertain or non-well-defined cancers.

Alcohol consumption categories are based on the average weekly intake of standard drinks relative to UK guidelines. In the UK, one standard drink equals to 8 g of pure alcohol. Within guidelines: ≤ 14 units/week; above guidelines:>14 units/week.

### Independent association of the exposures

Compared with the bottom % BF tertile, both middle and top tertiles showed increased risk across all cancer outcomes except total cancer, with no evidence of dose–response (online Supplementary Table 2). We found similar association between WC and BMI with cancer incidence (online Supplementary Tables 3 and 4). Alcohol consumption was associated with total cancer risk, but only above guideline drinking was associated with alcohol-related cancer risk. Current alcohol consumption was not associated with outcomes that included obesity-related cancer (online Supplementary Table 5).

### Joint association of alcohol and adiposity with incident cancer

#### Body fat percentage

**Fig. 1.**
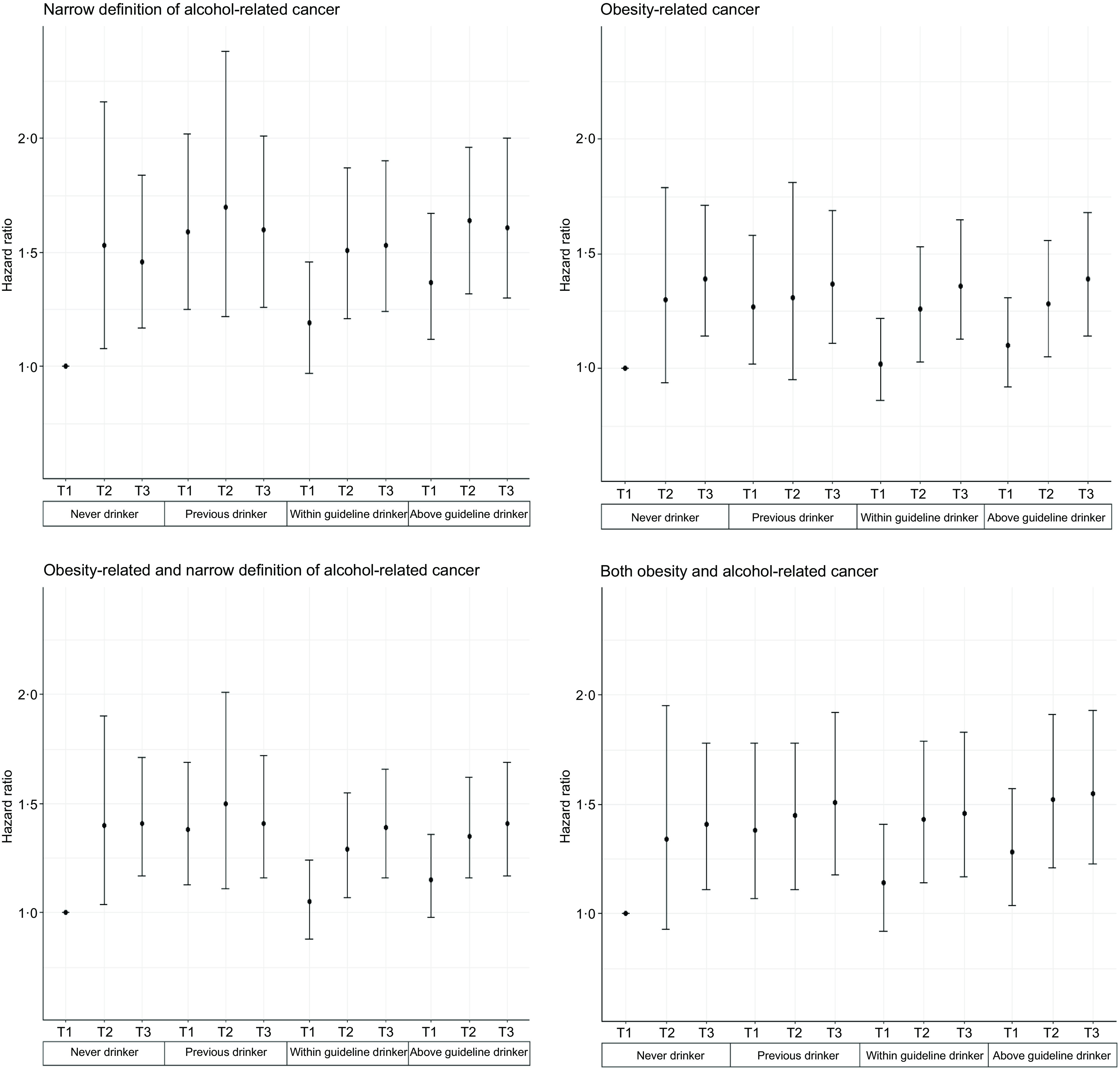
Joint associations between alcohol consumption and body fat percentage with cancer incidence (*n* 399 575). Cox proportional hazard model. Never drinker I 1^st^ T is the referent group. Model is adjusted for baseline age, sex, smoking status, dietary pattern score (determined by higher consumption of fruit, vegetables, and fish and lower consumption of processed meats and red meats (Rutten-Jacobs *et al.*, 2018)), sleep duration (h/night), education, Townsend Deprivation Index, physical activity ((MET)-hour/week) and chronic diseases (major CVD (ICD-10 codes I00 to I99), type 2 diabetes (ICD-10 codes E11·0 to E11·9 and E12) and dyslipidaemia (ICD-10 codes E78·0–E78·6) diagnosed by a doctor and hospital admission records and self-reported CVD and type 2 diabetes). Body fat percentage (BF %) was measured by bioimpedance using the Tanita BC-418MA device (Tanita). BF % by tertile: Tertile 1 (T1): <23·1 % for women and <33·9 % for men, Tertile 2 (T2): 23·1–27·8 % for women and 33·9–39·8 for men, Tertile 3 (T3): >27·8 % for women and >39·8 % for men. Alcohol consumption categories are based on the average weekly intake of standard drinks relative to UK guidelines. In the UK, one standard drink equals to 8 g of pure alcohol. Within guidelines: ≤ 14 units/week; above guidelines:>14 units/week. Alcohol-related cancer according to the narrow definition included oral cavity, throat, larynx, oesophagus, liver, colorectal, stomach and female breast (IARC, 2014). Obesity-related cancer included meningioma, multiple myeloma, adenocarcinoma of the oesophagus, and cancers of the thyroid, postmenopausal breast, gallbladder, stomach, liver, pancreas, kidney, ovaries, uterus, colon and rectum (colorectal) (Lauby-Secretan *et al.*, 2016).

Previous drinkers had the highest cancer risk across all analyses. Compared with the referent ‘optimal’ category (never drinker/bottom tertile BF %), most other groups had a higher risk of alcohol-related cancer (both narrow and broad definitions) (Fig. 1, online Supplementary Table 6). Participants in the middle and top BF % groups who drink within and above guideline levels had an elevated risk of alcohol-related cancers. Compared with the reference group, the risk of alcohol-related cancer for those in the top tertile BF % and within guideline drinkers was HR 1·53 (95 % CI (1·24, 1·90)) and for above guideline drinkers was HR 1·61 (95 % CI (1·30, 2·00)). The analysis of the joint association with the risk of combined cancers and overlapping cancers produced similar results (Fig. 1, online Supplementary Table 6). In addition, the risk of combined and overlapping cancers increased with elevated BF % in a dose–response manner.

Irrespective of alcohol consumption status, obesity-related cancer risk increased at higher BF % levels. Compared with the referent category, the top tertile BF % had higher risk of obesity-related cancer regardless of alcohol consumption. For example, never drinkers and above guideline drinkers who were in the top tertile BF % had a similar 39 % increased risk of obesity-related cancer (HR 1·39, 95 % CI (1·14, 1·71) and HR 1·39, 95 % CI (1·14, 1·68), respectively) (Fig. 1, online Supplementary Table 6). Alcohol consumption for all BF % categories was associated with total cancer incidence (online Supplementary Table 6).

#### Waist circumference

**Fig. 2.**
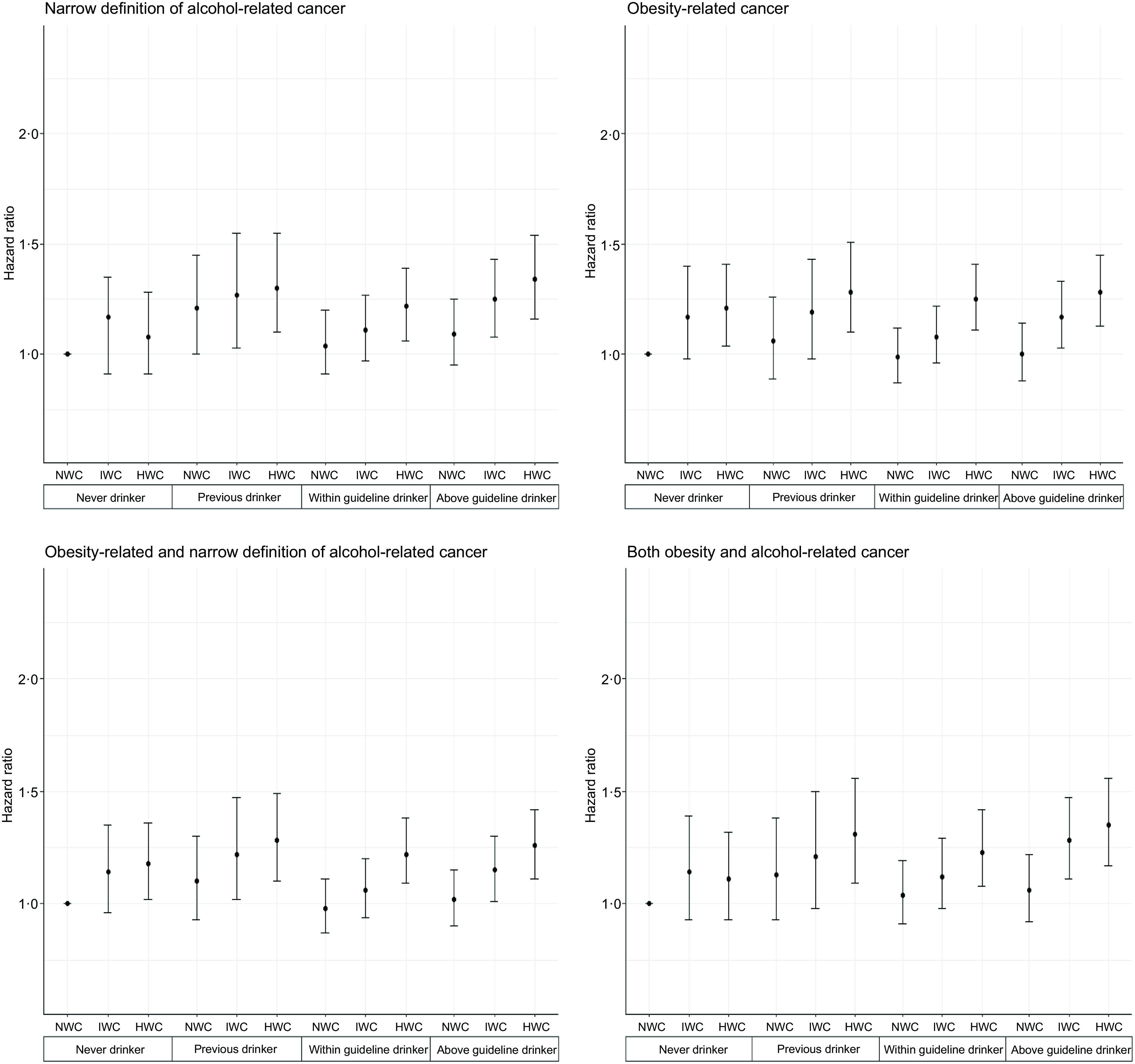
Joint associations between alcohol consumption and waist circumference with cancer incidence (*n* 399 575). Cox proportional hazard model. Never drinker I Normal WC is the referent group. Model is adjusted for baseline age, sex, smoking status, dietary pattern score (determined by higher consumption of fruit, vegetables, and fish and lower consumption of processed meats and red meats (Rutten-Jacobs *et al.*, 2018)), sleep duration (h/night), education, Townsend Deprivation Index, physical activity ((MET)-hour/week) and chronic diseases (major CVD (ICD-10 codes I00 to I99), type 2 diabetes (ICD-10 codes E11·0 to E11·9 and E12) and dyslipidaemia (ICD-10 codes E78·0–E78·6) diagnosed by a doctor and hospital admission records and self-reported CVD and type 2 diabetes). Waist circumference were measured by using flexible plastic tape with the participant in the resting-standing position by a trained professional. WHO classification: normal (<80 cm for women, <94 cm for men), increased risk of metabolic complications (80–88 cm for women, 94–102 cm for men), substantially increased risk of metabolic complications (>88 cm for women, >102 cm for men). NWC: normal waist circumference; IWC: Increased risk waist circumference; HWC: high-risk waist circumference. Alcohol consumption categories are based on the average weekly intake of standard drinks relative to UK guidelines. In the UK, one standard drink equals to 8 g of pure alcohol. Within guidelines: ≤ 14 units/week; above guidelines:>14 units/week. Alcohol-related cancer according to the narrow definition included oral cavity, throat, larynx, oesophagus, liver, colorectal, stomach and female breast (IARC, 2014). Obesity-related cancer included meningioma, multiple myeloma, adenocarcinoma of the oesophagus, and cancers of the thyroid, postmenopausal breast, gallbladder, stomach, liver, pancreas, kidney, ovaries, uterus, colon and rectum (colorectal) (Lauby-Secretan *et al*., 2016).

Risk for all cancer outcomes increased monotonically with elevated WC regardless of the level of alcohol consumption (Fig. 2, online Supplementary Table 7). For all WC groups, previous drinking was associated with alcohol-related cancer. In addition, among above guideline drinkers, participants with higher WC had a higher risk of alcohol-related cancer (HR 1·25, 95 % CI (1·08, 1·43) for increased risk of WC and HR 1·34, 95 % CI (1·16, 1·54) for high risk of WC) compared with the referent optimal category (never drinker/normal WC). Higher WC was associated with obesity-related cancer independent from alcohol consumption status. However, the risk increased with higher alcohol consumption (HR 1·21, 95 % CI (1·04, 1·41) for never drinkers; HR 1·25, 95 % CI (1·11, 1·42) for within guideline drinkers and HR 1·28, 95 % CI (1·10, 1·51) and HR 1·28, 95 % CI (1·13, 1·45) for above guideline drinkers). We observed a similar pattern of results for obesity-related cancer, as well as combined and overlapping cancers (online Supplementary Table 7).

#### BMI

**Fig. 3.**
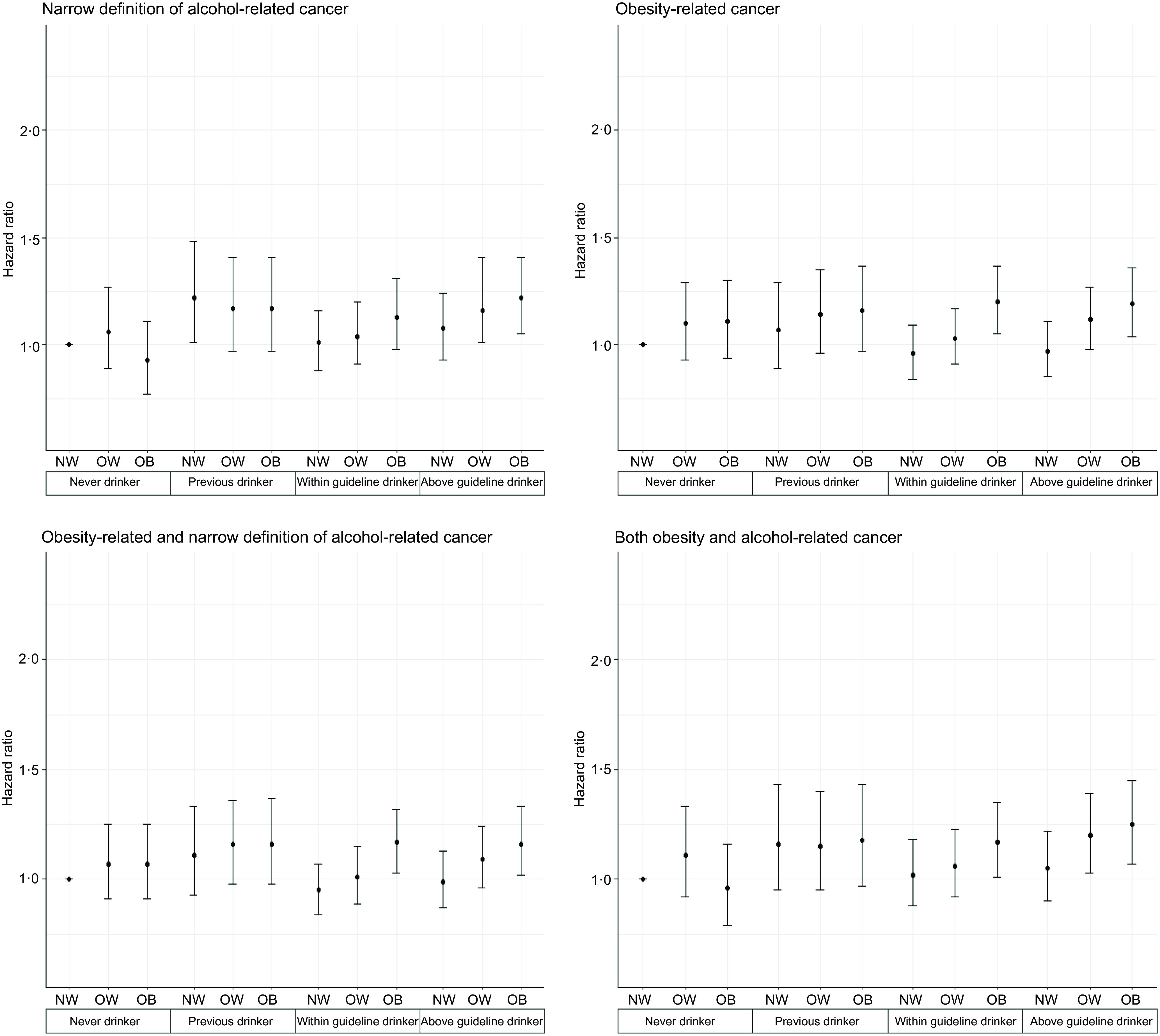
Joint associations between alcohol consumption and BMI with cancer incidence (*n* 399 575). Cox proportional hazard model. Never drinker I Normal weight is the referent group. Model is adjusted for baseline age, sex, smoking status, dietary pattern score (determined by higher consumption of fruit, vegetables, and fish and lower consumption of processed meats and red meats (Rutten-Jacobs *et al*., 2018)), sleep duration (h/night), education, Townsend Deprivation Index, physical activity ((MET)-h/week) and chronic diseases (major CVD (ICD-10 codes I00 to I99), Type 2 diabetes (ICD-10 codes E11·0 to E11·9 and E12) and dyslipidaemia (ICD-10 codes E78·0–E78·6) diagnosed by a doctor and hospital admission records and self-reported CVD and type 2 diabetes). BMI = Weight (kg)/height (m^2^). WHO classification: normal weight (18·5–24·9 kg/m^2^), overweight and obese (≥ 25·0 kg/m^2^). NW: normal weight; OW: overweight; OB: obese. Alcohol consumption categories are based on the average weekly intake of standard drinks relative to UK guidelines. In the UK, one standard drink equals to 8 g of pure alcohol. Within guidelines: ≤ 14 units/week; above guidelines:>14 units/week. Alcohol-related cancer according to the narrow definition included oral cavity, throat, larynx, oesophagus, liver, colorectal, stomach and female breast (IARC, 2014). Obesity-related cancer included meningioma, multiple myeloma, adenocarcinoma of the oesophagus, and cancers of the thyroid, postmenopausal breast, gallbladder, stomach, liver, pancreas, kidney, ovaries, uterus, colon and rectum (colorectal) (Lauby-Secretan *et al*., 2016).

The joint association of various levels of alcohol consumption and BMI with cancer (Fig. 3 and online Supplementary Table 8) was less clear than other adiposity markers. Only above guideline drinkers who were overweight or obese had higher alcohol-related cancer risk compared with the referent optimal group (HR 1·16, 95 % CI (1·01, 1·41) and HR 1·22, 95 % CI (1·05, 1·41), respectively). We also found that obesity was associated with a higher, but similar risk of obesity-related cancer among within guideline (HR 1·20, 95 % CI (1·05, 1·37)) and above guideline drinkers (HR 1·19, 95 % CI (1·04, 1·36)), with no evidence of dose–response. The joint association of alcohol consumption and BMI with risk of combined and overlapping cancer showed similar results. However, the point estimates were greater in overlapping cancer analyses than combined cancer analysis. For instance, above the guideline drinker and obese participants showed a slightly higher overlapping cancer risk (HR 1·25, 95 % CI (1·07, 1·45)) than combined cancer (HR 1·16, 95 % CI (1·02, 1·33)) (online Supplementary Table 8).

#### Sensitivity analysis

Exclusion of cases that occurred in the first 3 years of follow-up did not appreciably change the results for the joint association between alcohol consumption and adiposity markers with obesity- and alcohol-related cancer incidence (online Supplementary Table 9).

The joint association of alcohol consumption and adiposity markers with risk of each individual alcohol-related cancer site showed that participants who drink within guideline and above guideline had a higher risk of breast cancer across all WC and BMI groups, with some evidence of dose–response associations (online Supplementary Table 10).

We found statistically significant three-way interaction effects between smoking status, alcohol consumption and adiposity markers for most outcomes, for example, for alcohol-related cancer and total cancer (*P* < 0·05 for all adiposity markers) (online Supplementary Table 11). The joint association analysis between alcohol consumption and smoking with alcohol-related cancer incidence showed that current and previous alcohol consumption was associated with higher risk of alcohol-related cancer among previous and current smokers, but not never smokers (online Supplementary Table 12).

## Discussion

Our study is the first investigation into the joint association of alcohol consumption and multi-indicator adiposity with exposure-specific cancer incidence outcomes. We found that higher levels of adiposity modestly amplified the deleterious association of alcohol on cancer. These synergistic effects were more pronounced for BF %. We also showed that regardless of alcohol consumption status, higher adiposity levels increased the risk of obesity-related cancer.

Despite a weak independent association of alcohol consumption and cancer outcomes, we found some evidence of the deleterious effect of the joint association with higher adiposity. Across all markers of adiposity, above guideline drinkers who were in the top two adiposity groups had a higher cancer risk. The literature on the joint association of alcohol consumption and adiposity with cancer is limited to isolated cancer sites, such as liver cancer^(20,41)^, breast cancer^(42)^ and colorectal cancer^(21)^. Marrero *et al*.^(41)^ and Loomba *et al*.^(20)^ showed that alcohol and obesity synergistically increased the risk of hepatocellular carcinoma. While, Miller *et al.* ^(42)^ found ecological evidence linking obesity and alcohol consumption with breast cancer. In a case–control study of Zhao *et al*.^(21)^, the effect of alcohol consumption was associated with a higher risk of colorectal cancer only in the presence of obesity. Although previous studies suggested that alcohol consumption may independently increase the risk of certain cancers^(6,15,43–45)^, our study showed a higher risk of cancer only among above guideline drinkers with excess adiposity. While excess adiposity was usually considered as a potential confounder in previous studies investigating alcohol consumption and cancer risk^(6,45)^, our analyses examining how adiposity modified the effects of alcohol consumption on cancer risk offers more nuanced information. Our results, which reconcile several limitations in the literature, provide impetus for assessing interactions between alcohol and adiposity in future observational studies.

The elevated risk of alcohol-related cancers was mostly observed in participants with excess adiposity, suggesting a synergistic effect between these two modifiable risk factors. Such a synergistic effect can be explained by common mechanistic pathways of excess adiposity and alcohol consumption involved in carcinogenesis. In the case of liver cancer, for example, both obesity and alcohol consumption could lead to oxidative stress and liver injury via multiple metabolic pathways, including cytochrome P450 2E1 induction, free radical generation, lipid peroxidation, TNF-*α* production and increased transcription of pro-inflammatory mediators^(46–48)^. Additionally, our analyses on the joint association between alcohol consumption and adiposity markers with risk of individual alcohol-related cancer showed that the risk of breast cancer increased with elevated WC and BMI in a dose–response manner among alcohol drinkers, indicating a possible synergistic effect between alcohol consumption and adiposity. Alcohol consumption and obesity share common biological mechanisms in breast carcinogenesis through circulating sex hormone levels such as oestrogen and sex hormone-binding globulin^(49)^. The biological mechanisms explaining the joint effects of alcohol consumption and adiposity on cancer risk we have found require further investigation.

Our study showed that BMI has a certain value for assessing cancer risk, but it may not be the most suitable adiposity marker. Because BMI is an indirect measure of adiposity and does not differentiate between body fat and lean body mass, nor does it specify the location of adiposity^(50)^, WC is often used as a surrogate marker of body fat distribution. Although WC and BMI are strongly correlated with general adiposity, WC is a better predictor of visceral adipose tissue^(51)^. Excess visceral adipose tissue involves the metabolic pathways of systemic inflammation, insulin resistance and the activation of the insulin-like growth factor system, leading to a pro-tumourigenic microenvironment that promotes cancer development^(52,53)^. Previous studies on different cancer sites showed that WC and BF % had better discriminatory power than BMI in cancer risk^(54–57)^.

Previous study on the association between intentional weight change and obesity-related cancer incidence in the Women’s Health Initiative Observational Study demonstrated that intentional weight loss or WC reduction was associated with lower risk of obesity-related cancers^(58)^. A meta-analysis of nine case–control studies found that 5 years of alcohol cessation was associated with a reduction of 15 % in risk of laryngeal and pharyngeal cancers^(59)^. Our findings provide novel information to further develop alcohol consumption and overweight and obesity management guidelines as well as emphasise how alcohol consumption guidelines and clinical advice may need to consider upward trends of obesity and overweight prevalence. This body of evidence suggesting an association between excess adiposity and alcohol consumption with many cancers substantiates the potential value of reducing alcohol consumption and weight loss as a method of cancer risk reduction.

Alcohol consumption and smoking are both biologically plausible tumour-promoting lifestyle risk factors^(40)^. Previous research showed that alcohol drinkers and smokers have greater risk of cancer incidence than never drinkers and smokers, indicating possible synergistic effects^(60,61)^. We also found that previous and current smokers who currently drink alcohol or stopped drinking have higher risk of alcohol-related cancer. As healthy lifestyle factors can have a synergistic effect and offer substantial advantages over a single factor^(62)^, the association between adiposity, alcohol consumption and smoking with cancer risk merits further investigation.

### Strengths and limitations

This study has several strengths, including the large longitudinal sample and hard end points (hospital admission and cancer registry). We were able to provide detailed exposure-specific outcomes (alcohol- and obesity-related cancers as well as their combinations). We were able to adjust for extensive covariates such as diet, illness and socio-economic status. Measurement of adiposity markers by trained staff using standardised techniques minimised the chance of measurement error and misclassification. We also had detailed information on drinking volume. The study has some potential limitations. Firstly, we relied on self-reported alcohol consumption which may lead to underreporting and consequently reporting biases. Still, self-reported alcohol consumption measures have demonstrated adequate reliability and validity in previous studies^(63)^. Additionally, we had no information on when previous drinkers stopped drinking, nor the duration, quantity or frequency of their alcohol consumption before they stopped drinking. Previous drinkers may have given up alcohol consumption due to the occurrence of health conditions, some of which may be associated with future risk of cancer and other health outcomes. This has been referred to as the ‘sick-quitter effect’^(64)^ and could result in non-drinkers appearing to have higher cancer risk in comparison with light and moderate drinkers. We addressed this issue by using never drinkers as the referent group for alcohol consumption rather than using all non-drinkers combined. Residual confounding is also possible despite a comprehensive adjustment scheme. Although we used data from cancer registries, we cannot exclude misclassification for cancer-specific sites or uncommon cancers. In addition, we grouped all the known cancers related to alcohol and obesity which may result in missing cancer site-specific effects. The UK Biobank has very low response rate (5·5 %) and may be prone to healthy volunteer selection bias. However, Stamatakis *et al*.^(65)^ recently showed that poor representativeness does not affect materially the prospective association of alcohol with cancer risk.

### Conclusion

We found some evidence that excess adiposity may exacerbate the harmful effect of alcohol on cancer risk. Our study adds important evidence to understanding the joint role of adiposity markers and alcohol consumption in potentially leading to higher cancer incidence and supports combined alcohol consumption and adiposity reduction measures as clinical and public health priorities aimed at lowering cancer risk.

## Supplementary material

For supplementary material accompanying this paper visit https://doi.org/10.1017/S0007114522003464. click here to view supplementary material
