# Supplementary material for: Joint association of alcohol consumption and adiposity with alcohol- and obesity-related cancer in a population sample of 399,575 UK adults
Source: Br J Nutr. 2022 Oct 21;130(3):503–12. doi: 10.1017/S0007114522003464 (PMC10331437; doi:10.1017/S0007114522003464)
Supplement: Supplementary file 1 [file S0007114522003464sup001.docx]

**Supplementary Figure 1.** Participant recruitment flowchart

**Supplementary Table 1.** Interaction between alcohol consumption and adiposity markers for cancer incidence by various definitions of cancer (N=385,927)*

|  | Alcohol consumption*BF% | Alcohol consumption*WC | Alcohol consumption*BMI |
| --- | --- | --- | --- |
|  | *P* | *P* | *P* |
| **Narrow definition of alcohol-related cancer** | 0.613 | 0.867 | 0.134 |
| **Obesity-related cancer** | 0.813 | 0.455 | 0.103 |
| **Obesity-related & narrow definition of alcohol-related cancer (combined)** | 0.208 | 0.095 | 0.001 |
| **Both obesity and alcohol-related cancer (overlapping)** | 0.047 | 0.289 | 0.719 |
| **Broad definition of alcohol-related cancer** | 0.887 | 0.015 | 0.002 |
| **Total cancer** | 0.076 | <0.001 | <0.001 |
| *Previous drinkers were excluded from the analysis.  Model is adjusted for baseline age and sex.  Body Fat (BF%) was measured by bioimpedance using the Tanita BC-418MA device (Tanita, Tokyo, Japan). Waist circumference (WC) was measured by using flexible plastic tape with the participant in the resting-standing position by a trained professional. Body mass index (BMI) = Weight (kg)/height (m^2^). | | | |

**Supplementary Table 2.** Independent association between BF% and cancer incidence by various definitions of cancer (N=399,575)

|  | Events / n | HR (95% CI) |
| --- | --- | --- |
| **Narrow definition of alcohol-related cancer** | | |
| Bottom Tertile BF% | 5,704 / 173,601 | - |
| Middle Tertile BF% | 1,238 / 26,771 | *1.23 (1.13 – 1.33)* |
| Top Tertile BF% | 11,304 / 199,203 | *1.24 (1.13 – 1.35)* |
| **Obesity-related cancer** | | |
| Bottom Tertile BF% | 5,357 / 173,601 | - |
| Middle Tertile BF% | 1,319 / 26,771 | *1.19 (1.10 – 1.29)* |
| Top Tertile BF% | 13,448 / 199,203 | *1.29 (1.19 – 1.41)* |
| **Obesity-related & narrow definition of alcohol-related cancer (combined)** | | |
| Bottom Tertile BF% | 6,236 / 173,601 | - |
| Middle Tertile BF% | 1,447 / 26,771 | *1.20 (1.11 – 1.29)* |
| Top Tertile BF% | 13,887 / 199,203 | *1.27 (1.17 – 1.38)* |
| **Both obesity and alcohol-related cancer (overlapping)** | | |
| Bottom Tertile BF% | 4,368 / 173,601 | - |
| Middle Tertile BF% | 1,133 / 26,771 | 1.22 (1.12 – 1.33) |
| Top Tertile BF% | 10,979 / 199,203 | 1.25 (1.14 – 1.37) |
| **Broad definition of alcohol-related cancer** | | |
| Bottom Tertile BF% | 7,245 / 173,601 | - |
| Middle Tertile BF% | 1,568 / 26,771 | *1.20 (1.12 – 1.28)* |
| Top Tertile BF% | 13,398 / 199,203 | *1.19 (1.10 – 1.28)* |
| **Total cancer** | | |
| Bottom Tertile BF% | 30,181 / 173,601 | - |
| Middle Tertile BF% | 4,032 / 26,771 | 0.95 (0.91 – 0.98) |
| Top Tertile BF% | 27,685 / 199,203 | 0.91 (0.87 – 0.95) |
| Cox proportional hazard model. 1^st^ tertile is the referent group.  Model is adjusted for baseline age, sex, smoking status, dietary pattern score (determined by higher consumption of fruit, vegetables, and fish and lower consumption of processed meats and red meats (Rutten-Jacobs et al., 2018)), sleep duration (hrs/night), education, Townsend Deprivation Index, physical activity ((MET)-hour/week), total alcohol consumption unit, chronic diseases (major cardiovascular disease (ICD-10 codes I00 to I99), Type 2 diabetes (ICD-10 codes E11.0 to E11.9 and E12) and dyslipidaemia (ICD-10 codes E78.0-E78.6) diagnosed by a doctor and hospital admission records and self-reported cardiovascular diseases and Type 2 diabetes).  Body Fat (BF%) was measured by bioimpedance using the Tanita BC-418MA device (Tanita, Tokyo, Japan). BF% by tertile: Bottom Tertile: <23.1% for women and <33.9% for men, Middle Tertile: 23.1-27.8% for women and 33.9-39.8 for men, Top Tertile: >27.8% for women and >39.8% for men.  Alcohol-related cancer according to the broad definition included oral cavity, throat, larynx, esophagus, liver, colorectal, stomach, female breast, pancreas and lung cancer (IARC, 2014).  Alcohol-related cancer according to the narrow definition included oral cavity, throat, larynx, esophagus, liver, colorectal, stomach, female breast (IARC, 2014).  Obesity-related cancer included meningioma, multiple myeloma, adenocarcinoma of the esophagus, and cancers of the thyroid, postmenopausal breast, gallbladder, stomach, liver, pancreas, kidney, ovaries, uterus, colon and rectum (colorectal) (Lauby‑Secretan et al., 2016).  The definition of total cancer excludes in situ, benign, uncertain, or non-well-defined cancers.  *Italic font indicates statistical significance (p<0.05).* | | |

**Supplementary Table 3.** Independent association between WC and cancer incidence by various definitions of cancer (N=399,575)

|  | Events / n | HR (95% CI) |
| --- | --- | --- |
| **Narrow definition of alcohol-related cancer** | | |
| Normal WC | 6,342 / 169,832 | - |
| Increased risk WC | 4,684 / 105,175 | *1.09 (1.05 – 1.13)* |
| High risk WC | 6,590 / 124,568 | *1.18 (1.13 – 1.22)* |
| **Obesity-related cancer** | | |
| Normal WC | 6,967 / 169,832 | - |
| Increased risk WC | 5,303 / 105,175 | *1.12 (1.08 – 1.17)* |
| High risk WC | 7,854 / 124,568 | *1.27 (1.23 – 1.31)* |
| **Obesity-related & narrow definition of alcohol-related cancer (combined)** | | |
| Normal WC | 7,851 / 169,832 | *-* |
| Increased risk WC | 5,661 / 105,175 | *1.10 (1.06 – 1.14)* |
| High risk WC | 8,328 / 124,568 | *1.23 (1.19 – 1.27)* |
| **Both obesity and alcohol-related cancer (overlapping)** | | |
| Normal WC | 5,831 / 169,832 | - |
| Increased risk WC | 4,420 / 105,175 | *1.12 (1.08 – 1.17)* |
| High risk WC | 6,229 / 124,568 | *1.21 (1.16 – 1.26)* |
| **Broad definition of alcohol-related cancer** | | |
| Normal WC | 7,932 / 169,832 | - |
| Increased risk WC | 5,929 / 105,175 | *1.07 (1.03 – 1.10)* |
| High risk WC | 8,350 / 124,568 | *1.14 (1.10 – 1.18)* |
| **Total cancer** | | |
| Normal WC | 24,181 / 169,832 | - |
| Increased risk WC | 16,941 / 105,175 | 1.00 (0.98 – 1.02) |
| High risk WC | 20,776 / 124,568 | *1.02 (1.00 – 1.04)* |
| Cox proportional hazard model. Normal WC is the referent group.  Model is adjusted for baseline age, sex, smoking status, dietary pattern score (determined by higher consumption of fruit, vegetables, and fish and lower consumption of processed meats and red meats (Rutten-Jacobs et al., 2018)), sleep duration (hrs/night), education, Townsend Deprivation Index, physical activity ((MET)-hour/week), total alcohol consumption unit, chronic diseases (major cardiovascular disease (ICD-10 codes I00 to I99), Type 2 diabetes (ICD-10 codes E11.0 to E11.9 and E12) and dyslipidaemia (ICD-10 codes E78.0-E78.6) diagnosed by a doctor and hospital admission records and self-reported cardiovascular diseases and Type 2 diabetes).  Waist circumference was measured by using flexible plastic tape with the participant in the resting-standing position by a trained professional. WHO classification: normal (<80 cm for women, <94 cm for men), increased risk of metabolic complications (80-88 cm for women, 94-102 cm for men), substantially increased risk of metabolic complications (>88 cm for women, >102 cm for men).  Alcohol-related cancer according to the broad definition included oral cavity, throat, larynx, esophagus, liver, colorectal, stomach, female breast, pancreas and lung cancer (IARC, 2014).  Alcohol-related cancer according to the narrow definition included oral cavity, throat, larynx, esophagus, liver, colorectal, stomach, female breast (IARC, 2014).  Obesity-related cancer included meningioma, multiple myeloma, adenocarcinoma of the esophagus, and cancers of the thyroid, postmenopausal breast, gallbladder, stomach, liver, pancreas, kidney, ovaries, uterus, colon and rectum (colorectal) (Lauby‑Secretan et al., 2016).  The definition of total cancer excludes in situ, benign, uncertain, or non-well-defined cancers.  *Italic font indicates statistical significance (p<0.05).* | | |

**Supplementary Table 4.** Independent association between BMI and cancer incidence by various definitions of cancer (N=399,575)

|  | Events / n | HR (95% CI) |
| --- | --- | --- |
| **Narrow definition of alcohol-related cancer** | | |
| Normal weight | 5,459 / 130,284 | - |
| Overweight | 7,315 / 170,417 | *1.05 (1.01-1.08)* |
| Obese | 4,842 / 98,874 | *1.11 (1.07-1.16)* |
| **Obesity-related cancer** | | |
| Normal weight | 5,974 / 130,284 | - |
| Overweight | 8,310 / 170,417 | *1.10 (1.06 – 1.14)* |
| Obese | 5,840 / 98,874 | *1.24 (1.19 – 1.28)* |
| **Obesity-related & narrow definition of alcohol-related cancer (combined)** | | |
| Normal weight | 6,433 / 130,284 | *-* |
| Overweight | 8,926 / 170,417 | *1.08 (1.04, 1.11)* |
| Obese | 6,211 / 98,874 | *1.19 (1.15, 1.24)* |
| **Both obesity and alcohol-related cancer (overlapping)** | | |
| Normal weight | 5,071 / 130,284 | - |
| Overweight | 6,854 / 170,417 | 1.08 (1.04 – 1.12) |
| Obese | 4,555 / 98,874 | 1.14 (1.10 – 1.19) |
| **Broad definition of alcohol-related cancer** | | |
| Normal weight | 6,799 / 130,284 | - |
| Overweight | 9,315 / 170,417 | 1.01 (0.98 – 1.05) |
| Obese | 6,097 / 98,874 | *1.05 (1.01 – 1.09)* |
| **Total cancer** | | |
| Normal weight | 18,592 / 130,284 | - |
| Overweight | 27,484 / 170,417 | 0.99 (0.97 – 1.01) |
| Obese | 15,822 / 98,874 | 0.97 (0.95 – 1.00) |
| Cox proportional hazard model. Normal weight is the referent group.  Model is adjusted for baseline age, sex, smoking status, dietary pattern score (determined by higher consumption of fruit, vegetables, and fish and lower consumption of processed meats and red meats (Rutten-Jacobs et al., 2018)), sleep duration (hrs/night), education, Townsend Deprivation Index, physical activity ((MET)-hour/week), total alcohol consumption unit, chronic diseases (major cardiovascular disease (ICD-10 codes I00 to I99), Type 2 diabetes (ICD-10 codes E11.0 to E11.9 and E12) and dyslipidaemia (ICD-10 codes E78.0-E78.6) diagnosed by a doctor and hospital admission records and self-reported cardiovascular diseases and Type 2 diabetes). Body mass index = Weight (kg)/height (m^2^). WHO classification: normal weight (18.5-24.9 kg/m^2^), overweight and obese (≥ 25.0 kg/m^2^).  Alcohol-related cancer according to the broad definition included oral cavity, throat, larynx, esophagus, liver, colorectal, stomach, female breast, pancreas and lung cancer (IARC, 2014).  Alcohol-related cancer according to the narrow definition included oral cavity, throat, larynx, esophagus, liver, colorectal, stomach, female breast (IARC, 2014).  Obesity-related cancer included meningioma, multiple myeloma, adenocarcinoma of the esophagus, and cancers of the thyroid, postmenopausal breast, gallbladder, stomach, liver, pancreas, kidney, ovaries, uterus, colon and rectum (colorectal) (Lauby‑Secretan et al., 2016).  The definition of total cancer excludes in situ, benign, uncertain, or non-well-defined cancers.  *Italic font indicates statistical significance (p<0.05).* | | |

**Supplementary Table 5.** Independent association between alcohol consumption and cancer (N=399,575)

|  | Events / n | HR (95% CI) |
| --- | --- | --- |
| **Narrow definition of alcohol-related cancer** | | |
| Never drinker | 742 / 17,061 | - |
| Previous drinker | 691 / 13,648 | *1.19 (1.07 – 1.32)* |
| Within guidelines drinker | 10,072 / 224,171 | 1.07 (0.99 – 1.15) |
| Above the guidelines drinker | 6,111 / 144,695 | *1.16 (1.07 – 1.26)* |
| **Obesity-related cancer** |  |  |
| Never drinker | 962/ 17,061 | - |
| Previous drinker | 755 / 13,648 | 1.05 (0.95 -1.15) |
| Within guidelines drinker | 11,832 / 224,171 | 1.00 (0.93 – 1.06) |
| Above the guidelines drinker | 6,575 / 144,695 | 1.04 (0.97 – 1.11) |
| **Obesity-related & narrow definition of alcohol-related cancer (combined)** | | |
| Never drinker | 1006 / 17,061 | *-* |
| Previous drinker | 843 / 13,648 | 1.09 (0.99 – 1.19) |
| Within guideline drinker | 12,466 / 224,171 | 0.99 (0.92 – 1.05) |
| Above the guideline drinker | 7,255 / 144,695 | 1.03 (0.96 – 1.10) |
| **Both obesity and alcohol-related cancer (overlapping)** | | |
| Never drinker | 717 / 17,061 | - |
| Previous drinker | 619 / 13,648 | *1.14 (1.02 – 1.27)* |
| Within guideline drinker | 9,588 / 224,171 | 1.03 (0.95 – 1.11) |
| Above the guideline drinker | 5,556 / 144,695 | 0.99 (0.91 – 1.08) |
| **Broad definition of alcohol-related cancer** | | |
| Never drinker | 922 / 17,061 | - |
| Previous drinker | 966 / 13,648 | *1.21 (1.11 – 1.33)* |
| Within guideline drinker | 12,362 / 224,171 | 1.04 (0.98 – 1.12) |
| Above the guideline drinker | 7,961 / 144,695 | *1.10 (1.04 – 1.19)* |
| **Total cancer** | | |
| Never drinker | 2,229 / 17,061 | - |
| Previous drinker | 2,215 / 13,648 | *1.15 (1.09 – 1.22)* |
| Within guideline drinker | 33,362 / 224,171 | *1.13 (1.09 – 1.18)* |
| Above the guideline drinker | 24,092 / 144,695 | *1.14 (1.09 – 1.19)* |
| Cox proportional hazard model. Never drinker is the referent group.  Model is adjusted for baseline age, sex, smoking status, dietary pattern score (determined by higher consumption of fruit, vegetables, and fish and lower consumption of processed meats and red meats (Rutten-Jacobs et al., 2018)), sleep duration (hrs/night), education, Townsend Deprivation Index, physical activity ((MET)-hour/week), BMI (kg/m^2^), chronic diseases (major cardiovascular disease (ICD-10 codes I00 to I99), Type 2 diabetes (ICD-10 codes E11.0 to E11.9 and E12) and dyslipidaemia (ICD-10 codes E78.0-E78.6) diagnosed by a doctor and hospital admission records and self-reported cardiovascular diseases and Type 2 diabetes).  Alcohol consumption categories are based on the average weekly intake of standard drinks relative to UK guidelines. In the UK, one standard drink equals to 8 g of pure alcohol. Within guidelines: ≤ 14 units/week; above guidelines:>14 units/week.  Alcohol-related cancer according to the broad definition included oral cavity, throat, larynx, esophagus, liver, colorectal, stomach, female breast, pancreas and lung cancer (IARC, 2014).  Alcohol-related cancer according to the narrow definition included oral cavity, throat, larynx, esophagus, liver, colorectal, stomach, female breast (IARC, 2014).  Obesity-related cancer included meningioma, multiple myeloma, adenocarcinoma of the esophagus, and cancers of the thyroid, postmenopausal breast, gallbladder, stomach, liver, pancreas, kidney, ovaries, uterus, colon and rectum (colorectal) (Lauby‑Secretan et al., 2016).  The definition of total cancer excludes in situ, benign, uncertain, or non-well-defined cancers.  *Italic font indicates statistical significance (p<0.05).* | | |

**Supplementary Table 6.** Joint association between alcohol consumption and BF% with cancer incidence by various definitions of cancer (N=399,575)

|  | Events / n | Model 1  HR (95% CI) | Model 2  HR (95% CI) | Model 3  HR (95% CI) |
| --- | --- | --- | --- | --- |
| **Narrow definition of alcohol-related cancer** | | | | |
| **Never drinker** |  |  |  |  |
| Bottom Tertile BF% | 100 / 4,689 | *-* | *-* | *-* |
| Middle Tertile BF% | 48 / 1,010 | *1.63 (1.15, 2.31)* | *1.60 (1.13, 2.27)* | *1.53 (1.08, 2.16)* |
| Top Tertile BF% | 594 / 11,362 | *1.65 (1.32, 2.08)* | *1.61 (1.28, 2.02)* | *1.46 (1.17, 1.84)* |
| **Previous drinker** |  |  |  |  |
| Bottom Tertile BF% | 208 / 5,591 | *1.72 (1.36, 2.18)* | *1.60 (1.26, 2.03)* | *1.59 (1.25, 2.02)* |
| Middle Tertile BF% | 53 / 1,044 | *1.92 (1.37, 2.68)* | *1.77 (1.26, 2.47)* | *1.70 (1.22, 2.38)* |
| Top Tertile BF% | 430 / 7,013 | *1.93 (1.63, 2.43)* | *1.75 (1.39, 1.21)* | *1.60 (1.26, 2.01)* |
| **Within guideline** |  |  |  |  |
| Bottom Tertile BF% | 1,929 / 74,684 | 1.23 (1.00, 1.50) | 1.18 (0.96, 1.44) | 1.19 (0.97, 1.46) |
| Middle Tertile BF% | 678 / 15,397 | *1.66 (1.34, 2.07)* | *1.56 (1.25, 1.93)* | *1.51 (1.21, 1.87)* |
| Top Tertile BF% | 7,465 / 134,090 | *1.78 (1.44, 2.20)* | *1.65 (1.33, 2.04)* | *1.53 (1.24, 1.90)* |
| **Above guideline** |  |  |  |  |
| Bottom Tertile BF% | 2,837 / 88,637 | *1.47 (1.21, 1.80)* | *1.36 (1.11, 1.66)* | *1.37 (1.12, 1.67)* |
| Middle Tertile BF% | 459 / 9,320 | *1.88 (1.51, 2.34)* | *1.70 (1.37, 2.12)* | *1.64 (1.32, 1.96)* |
| Top Tertile BF% | 2,815 / 46,738 | *1.93 (1.56, 2.40)* | *1.72 (1.39, 2.14)* | *1.61 (1.30, 2.00)* |
| **Obesity-related cancer** | | | | |
| **Never drinker** |  |  |  |  |
| Bottom Tertile BF% | 130 / 4,689 | *-* | *-* | *-* |
| Middle Tertile BF% | 55 / 1,010 | *1.38 (1.00, 1.91)* | *1.37 (0.99, 1.89)* | 1.30 (0.94, 1.79) |
| Top Tertile BF% | 777 / 11,362 | *1.57 (1.29, 1.93)* | *1.55 (1.27, 1.90)* | *1.39 (1.14, 1.71)* |
| **Previous drinker** |  |  |  |  |
| Bottom Tertile BF% | 209 / 5,591 | *1.33 (1.07, 1.65)* | 1.28 (1.02, 1.59) | 1.27 (1.02, 1.58) |
| Middle Tertile BF% | 53 / 1,044 | *1.43 (1.04, 1.98)* | *1.37 (0.99, 1.89)* | 1.31 (0.95, 1.81) |
| Top Tertile BF% | 493 / 7,013 | *1.61 (1.31, 1.99)* | *1.53 (1.24, 1.88)* | *1.37 (1.11, 1.69)* |
| **Within guideline** |  |  |  |  |
| Bottom Tertile BF% | 2,128 / 74,684 | 1.03 (0.87, 1.24) | 1.01 (0.84, 1.20) | 1.02 (0.86, 1.22) |
| Middle Tertile BF% | 748 / 15,397 | *1.36 (1.12, 1.66)* | *1.30 (1.07, 1.58)* | 1.26 (1.03, 1.53) |
| Top Tertile BF% | 8,956 / 134,090 | *1.56 (1.29, 1.88)* | *1.48 (1.22, 1.79)* | *1.36 (1.13, 1.65)* |
| **Above guideline** |  |  |  |  |
| Bottom Tertile BF% | 2,890 / 88,637 | 1.15 (0.97, 1.38) | 1.09 (0.92, 1.30) | 1.10 (0.92, 1.31) |
| Middle Tertile BF% | 463 / 9,320 | *1.43 (1.17, 1.74)* | *1.33 (1.09, 1.63)* | *1.28 (1.05, 1.56)* |
| Top Tertile BF% | 3,222 / 46,738 | *1.62 (1.34, 1.97)* | *1.50 (1.24, 1.82)* | *1.39 (1.14, 1.68)* |
| **Obesity-related & narrow definition of alcohol-related cancer (combined)** | | | | |
| **Never drinker** |  |  |  |  |
| Bottom Tertile BF% | 145 / 4,689 | *-* | *-* | *-* |
| Middle Tertile BF% | 62 / 1,010 | *1.50 (1.11, 2.03)* | *1.48 (1.10, 2.00)* | *1.40 (1.04, 1.90)* |
| Top Tertile BF% | 799 / 11,362 | *1.61 (1.33, 1.95)* | *1.58 (1.30, 1.91)* | *1.41 (1.17, 1.71)* |
| **Previous drinker** |  |  |  |  |
| Bottom Tertile BF% | 258 / 5,591 | *1.47 (1.20, 1.80)* | *1.39 (1.13, 1.70)* | *1.38 (1.13, 1.69)* |
| Middle Tertile BF% | 65 / 1,044 | *1.66 (1.24, 2.23)* | *1.56 (1.16, 2.10)* | *1.50 (1.11, 2.01)* |
| Top Tertile BF% | 520 / 7,013 | *1.67 (1.38, 2.06)* | *1.57 (1.29, 1.92)* | *1.41 (1.16, 1.72)* |
| **Within guideline** |  |  |  |  |
| Bottom Tertile BF% | 2,425 / 74,684 | 1.06 (0.90, 1.25) | 1.03 (0.87, 1.22) | 1.05 (0.88, 1.24) |
| Middle Tertile BF% | 798 / 15,397 | *1.40 (1.17, 1.68)* | *1.34 (1.11, 1.61)* | *1.29 (1.07, 1.55)* |
| Top Tertile BF% | 9,243 / 134,090 | *1.59 (1.33, 1.91)* | *1.51 (1.26, 1.80)* | *1.39 (1.16, 1.66)* |
| **Above guideline** |  |  |  |  |
| Bottom Tertile BF% | 3,408 / 88,637 | *1.21 (1.03, 1.43)* | 1.14 (0.97, 1.35) | 1.15 (0.98, 1.36) |
| Middle Tertile BF% | 522 / 9,320 | *1.51 (1.25, 1.82)* | *1.40 (1.16, 1.69)* | *1.35 (1.16, 1.62)* |
| Top Tertile BF% | 3,325 / 46,738 | *1.66 (1.38, 1.99)* | *1.52 (1.27, 1.82)* | *1.41 (1.17, 1.69)* |
| **Both obesity and alcohol-related cancer (overlapping)** | | | | |
| **Never drinker** |  |  |  |  |
| Bottom Tertile BF% | 92 / 4,689 | *-* | *-* | *-* |
| Middle Tertile BF% | 42 / 1,010 | 1.43 (0.99, 2.08) | 1.41 (0.97, 2.04) | 1.34 (0.93, 1.95) |
| Top Tertile BF% | 583 / 11,362 | *1.58 (1.25, 2.00)* | *1.54 (1.22, 1.96)* | *1.41 (1.11, 1.78)* |
| **Previous drinker** |  |  |  |  |
| Bottom Tertile BF% | 163 / 5,591 | *1.47 (1.14, 1.90)* | *1.38 (1.07, 1.79)* | *1.38 (1.07, 1.78)* |
| Middle Tertile BF% | 47 / 1,044 | *1.75 (1.22, 2.49)* | *1.63 (1.15, 2.33)* | *1.58 (1.11, 1.78)* |
| Top Tertile BF% | 409 / 7,013 | *1.79 (1.41, 2.28)* | *1.65 (1.30, 2.11)* | *1.51 (1.18, 1.92)* |
| **Within guideline** |  |  |  |  |
| Bottom Tertile BF% | 1,700 / 74,684 | 1.17 (0.95, 1.45) | 1.13 (0.91, 1.39) | 1.14 (0.92, 1.41) |
| Middle Tertile BF% | 637 / 15,397 | *1.57 (1.25, 1.97)* | *1.47 (1.17, 1.85)* | *1.43 (1.14, 1.79)* |
| Top Tertile BF% | 7,251 / 134,090 | *1.68 (1.35, 2.10)* | *1.57 (1.25, 1.96)* | *1.46 (1.17, 1.83)* |
| **Above guideline** |  |  |  |  |
| Bottom Tertile BF% | 2,413 / 88,637 | *1.37 (1.11, 1.68)* | *1.27 (1.03, 1.56)* | *1.28 (1.04, 1.57)* |
| Middle Tertile BF% | 407 / 9,320 | *1.72 (1.47, 2.30)* | *1.57 (1.25, 1.96)* | *1.52 (1.21, 1.91)* |
| Top Tertile BF% | 2,736 / 46,738 | *1.84 (1.47, 2.30)* | *1.65 (1.32, 2.07)* | *1.55 (1.23, 1.93)* |
| **Broad definition of alcohol-related cancer** | | | | |
| **Never drinker** |  |  |  |  |
| Bottom Tertile BF% | 144 / 4,689 | *-* | *-* | *-* |
| Middle Tertile BF% | 59 / 1,010 | *1.56 (1.15, 2.12)* | *1.51 (1.11, 2.05)* | *1.43 (1.06, 1.95)* |
| Top Tertile BF% | 719 / 11,362 | *1.63 (1.35, 1.98)* | *1.53 (1.26, 1.86)* | *1.38 (1.14, 1.68)* |
| **Previous drinker** |  |  |  |  |
| Bottom Tertile BF% | 329 / 5,591 | *1.87 (1.54, 2.72)* | *1.59 (1.31, 1.94)* | *1.58 (1.30, 1.93)* |
| Middle Tertile BF% | 79 / 1,044 | *2.14 (1.62, 2.82)* | *1.81 (1.37, 2.39)* | *1.73 (1.31, 2.28)* |
| Top Tertile BF% | 558 / 7,013 | *2.05 (1.69, 2.50)* | *1.67 (1.37, 2.03)* | *1.51 (1.24, 1.84)* |
| **Within guideline** |  |  |  |  |
| Bottom Tertile BF% | 2,759 / 74,684 | 1.21 (1.02, 1.43) | 1.15 (0.97, 1.36) | 1.16 (0.98, 1.38) |
| Middle Tertile BF% | 837 / 15,397 | *1.63 (1.36, 1.96)* | *1.48 (1.23, 1.77)* | *1.43 (1.19, 1.71)* |
| Top Tertile BF% | 8,766 / 134,090 | *1.72 (1.44, 2.06)* | *1.52 (1.27, 1.83)* | *1.41 (1.18, 1.69)* |
| **Above guideline** |  |  |  |  |
| Bottom Tertile BF% | 4,013 / 88,637 | *1.43 (1.21, 1.68)* | *1.27 (1.07, 1.49)* | *1.27 (1.08, 1.51)* |
| Middle Tertile BF% | 593 / 9,320 | *1.82 (1.52, 2.19)* | *1.55 (1.29, 1.87)* | *1.49 (1.24, 1.80)* |
| Top Tertile BF% | 3,355 / 46,738 | *1.92 (1.60, 2.31)* | *1.60 (1.33, 1.92)* | *1.49 (1.24, 1.78)* |
| **Total cancer** |  |  |  |  |
| **Never drinker** |  |  |  |  |
| Bottom Tertile BF% | 618 / 4,689 | *-* | *-* | *-* |
| Middle Tertile BF% | 143 / 1,010 | 1.10 (0.92, 1.32) | 1.10 (0.91, 1.32) | 1.06 (0.88, 1.23) |
| Top Tertile BF% | 1,468 / 11,362 | *1.11 (1.00, 1.23)* | *1.11 (1.00, 1.23)* | 1.02 (0.92, 1.14) |
| **Previous drinker** |  |  |  |  |
| Bottom Tertile BF% | 1,002 / 5,591 | *1.31 (1.18, 1.45)* | *1.26 (1.14, 1.39)* | *1.26 (1.14, 1.39)* |
| Middle Tertile BF% | 182 / 1,044 | *1.32 (1.11, 1.55)* | *1.28 (1.08, 1.51)* | *1.23 (1.04, 1.46)* |
| Top Tertile BF% | 1,031 / 7,013 | *1.26 (1.13, 1.41)* | *1.22 (1.09, 1.36)* | *1.13 (1.01, 1.26)* |
| **Within guideline** |  |  |  |  |
| Bottom Tertile BF% | 12,550 / 74,684 | *1.27 (1.17, 1.37)* | *1.22 (1.12, 1.33)* | *1.23 (1.14, 1.34)* |
| Middle Tertile BF% | 2,192 / 15,397 | *1.27 (1.16, 1.37)* | *1.23 (1.12, 1.35)* | *1.19 (1.08, 1.31)* |
| Top Tertile BF% | 18,620 / 134,090 | *1.23 (1.12, 1.35)* | *1.18 (1.08, 1.30)* | *1.12 (1.02, 1.23)* |
| **Above guideline** |  |  |  |  |
| Bottom Tertile BF% | 16,011 / 88,637 | *1.30 (1.20, 1.41)* | *1.23 (1.13, 1.33)* | *1.23 (1.14, 1.34)* |
| Middle Tertile BF% | 1,515 / 9,320 | *1.23 (1.12, 1.35)* | *1.16 (1.06, 1.28)* | *1.13 (1.02, 1.24)* |
| Top Tertile BF% | 6,566 / 46,738 | *1.28 (1.16, 1.40)* | *1.20 (1.09, 1.32)* | *1.14 (1.03, 1.25)* |
| Cox proportional hazard model. Never drinker I 1^st^ tertile is the referent group.  Model 1 is adjusted for baseline age and sex. Model 2 is additionally adjusted for smoking status, dietary pattern score (determined by higher consumption of fruit, vegetables, and fish and lower consumption of processed meats and red meats (Rutten-Jacobs et al., 2018)), sleep duration (hrs/night), education, Townsend Deprivation Index and physical activity ((MET)-hour/week). Model 3 is further adjusted for chronic diseases (major cardiovascular disease (ICD-10 codes I00 to I99), Type 2 diabetes (ICD-10 codes E11.0 to E11.9 and E12) and dyslipidaemia (ICD-10 codes E78.0-E78.6) diagnosed by a doctor and hospital admission records and self-reported cardiovascular diseases and Type 2 diabetes).  Body Fat (BF%) was measured by bioimpedance using the Tanita BC-418MA device (Tanita, Tokyo, Japan). BF% by tertile: Bottom Tertile 1: <23.1% for women and <33.9% for men, Middle Tertile: 23.1-27.8% for women and 33.9-39.8 for men, Top Tertile: >27.8% for women and >39.8% for men.  Alcohol consumption categories are based on the average weekly intake of standard drinks relative to UK guidelines. In the UK, one standard drink equals to 8 g of pure alcohol. Within guidelines: ≤ 14 units/week; above guidelines:>14 units/week.  Alcohol-related cancer according to the broad definition included oral cavity, throat, larynx, esophagus, liver, colorectal, stomach, female breast, pancreas and lung cancer (IARC, 2014).  Alcohol-related cancer according to the narrow definition included oral cavity, throat, larynx, esophagus, liver, colorectal, stomach, female breast (IARC, 2014).  Obesity-related cancer included meningioma, multiple myeloma, adenocarcinoma of the esophagus, and cancers of the thyroid, postmenopausal breast, gallbladder, stomach, liver, pancreas, kidney, ovaries, uterus, colon and rectum (colorectal) (Lauby‑Secretan et al., 2016).  The definition of total cancer excludes in situ, benign, uncertain, or non-well-defined cancers.  *Italic font indicates statistical significance (p<0.05).* | | | | |

**Supplementary Table 7.** Joint association between alcohol consumption and WC with cancer incidence by various definitions of cancer (N=399,575)

|  | Events / n | Model 1  HR (95% CI) | Model 2  HR (95% CI) | Model 3  HR (95% CI) |
| --- | --- | --- | --- | --- |
| **Narrow definition of alcohol-related cancer** | | | | |
| **Never drinker** |  |  |  |  |
| Normal WC | 227 / 6,195 | *-* | *-* | *-* |
| Increased Risk WC | 177 / 4,070 | 1.15 (0.94, 1.40) | 1.14 (0.94, 1.39) | 1.17 (0.91, 1.35) |
| High Risk WC | 338 / 6,796 | 1.19 (1.00, 1.40) | 1.17 (0.99, 1.39) | 1.08 (0.91, 1.28) |
| **Previous drinker** |  |  |  |  |
| Normal WC | 221 / 5,040 | *1.29 (1.07, 1.56)* | *1.21 (1.00, 1.45)* | *1.21 (1.00, 1.45)* |
| Increased Risk WC | 158 / 3,162 | *1.41 (1.15, 1.72)* | *1.31 (1.07, 1.61)* | *1.27 (1.03, 1.55)* |
| High Risk WC | 312 / 5,446 | *1.52 (1.28, 1.80)* | *1.41 (1.19, 1.67)* | *1.30 (1.10, 1.55)* |
| **Within guideline** |  |  |  |  |
| Normal WC | 3,687 / 96,709 | 1.08 (0.94, 1.23) | 1.03 (0.90, 1.18) | 1.04 (0.91, 1.20) |
| Increased Risk WC | 2,555 / 56,605 | *1.19 (1.04, 1.37)* | 1.13 (0.99, 1.29) | 1.11 (0.97, 1.27) |
| High Risk WC | 3,830 / 70,857 | *1.38 (1.21, 1.58)* | *1.29 (1.13, 1.48)* | *1.22 (1.06, 1.39)* |
| **Above guideline** |  |  |  |  |
| Normal WC | 2,207 / 61,888 | *1.17 (1.02, 1.35)* | 1.08 (0.94, 1.24) | 1.09 (0.95, 1.25) |
| Increased Risk WC | 1,794 / 41,338 | *1.39 (1.21, 1.60)* | *1.27 (1.11, 1.46)* | *1.25 (1.08, 1.43)* |
| High Risk WC | 2,110 / 41,469 | *1.57 (1.37, 1.80)* | *1.42 (1.23, 1.63)* | *1.34 (1.16, 1.54)* |
| **Obesity-related cancer** | | | | |
| **Never drinker** |  |  |  |  |
| Normal WC | 274 / 6,195 | *-* | *-* | *-* |
| Increased Risk WC | 226 / 4,070 | *1.21 (1.01, 1.44)* | *1.21 (1.02, 1.44)* | 1.17 (0.98, 1.40) |
| High Risk WC | 462 / 6,796 | *1.32 (1.14, 1.54)* | *1.33 (1.14, 1.54)* | *1.21 (1.04, 1.41)* |
| **Previous drinker** |  |  |  |  |
| Normal WC | 224 / 5,040 | 1.10 (0.92, 1.31) | 1.06 (0.89, 1.26) | 1.06 (0.89, 1.26) |
| Increased Risk WC | 172 / 3,162 | *1.28 (1.06, 1.55)* | *1.23 (1.02, 1.49)* | 1.19 (0.98, 1.43) |
| High Risk WC | 359 / 5,446 | *1.45 (1.24, 1.69)* | *1.40 (1.20, 1.66)* | *1.28 (1.10, 1.51)* |
| **Within guideline** |  |  |  |  |
| Normal WC | 4,145 / 96,709 | 1.01 (0.89, 1.14) | 0.97 (0.86, 1.10) | 0.99 (0.87, 1.12) |
| Increased Risk WC | 2,972 / 56,605 | *1.15 (1.01, 1.30)* | 1.11 (0.98, 1.25) | 1.08 (0.96, 1.22) |
| High Risk WC | 4,715 / 70,857 | *1.40 (1.24, 1.58)* | *1.34 (1.19, 1.52)* | *1.25 (1.11, 1.42)* |
| **Above guideline** |  |  |  |  |
| Normal WC | 2,324 / 61,888 | 1.05 (0.93, 1.20) | 0.99 (0.88, 1.13) | 1.00 (0.88, 1.14) |
| Increased Risk WC | 1,933 / 41,338 | *1.28 (1.13, 1.45)* | *1.20 (1.06, 1.36)* | *1.17 (1.03, 1.33)* |
| High Risk WC | 2,318 / 41,469 | *1.46 (1.29, 1.66)* | *1.37 (1.20, 1.55)* | *1.28 (1.13, 1.45)* |
| **Obesity-related & narrow definition of alcohol-related cancer (combined)** | | | | |
| **Never drinker** |  |  |  |  |
| Normal WC | 293 / 6,195 | *-* | *-* | *-* |
| Increased Risk WC | 235 / 4,070 | 1.18 (0.99, 1.40) | 1.18 (0.99, 1.40) | 1.14 (0.96, 1.35) |
| High Risk WC | 478 / 6,796 | *1.30 (1.12, 1.50)* | *1.29 (1.11, 1.49)* | *1.18 (1.02, 1.36)* |
| **Previous drinker** |  |  |  |  |
| Normal WC | 256 / 5,040 | 1.16 (0.98, 1.37) | 1.10 (0.93, 1.30) | 1.10 (0.93, 1.30) |
| Increased Risk WC | 195 / 3,162 | *1.34 (1.12, 1.60)* | *1.27 (1.06, 1.53)* | *1.22 (1.02, 1.47)* |
| High Risk WC | 392 / 5,446 | *1.47 (1.27, 1.72)* | *1.40 (1.20, 1.63)* | *1.28 (1.10, 1.49)* |
| **Within guideline** |  |  |  |  |
| Normal WC | 4,415 / 96,709 | 1.00 (0.89, 1.13) | 0.97 (0.86, 1.09) | 0.98 (0.87, 1.11) |
| Increased Risk WC | 3,126 / 56,605 | *1.13 (1.00, 1.27)* | 1.08 (0.96, 1.22) | 1.06 (0.94, 1.20) |
| High Risk WC | 4,925 / 70,857 | *1.37 (1.22, 1.54)* | *1.31 (1.16, 1.47)* | *1.22 (1.09, 1.38)* |
| **Above guideline** |  |  |  |  |
| Normal WC | 2,617 / 61,888 | 1.08 (0.95, 1.21) | 1.01 (0.89, 1.14) | 1.02 (0.90, 1.15) |
| Increased Risk WC | 2,105 / 41,338 | *1.26 (1.11, 1.42)* | *1.17 (1.04, 1.33)* | *1.15 (1.01, 1.30)* |
| High Risk WC | 2,533 / 41,469 | *1.45 (1.28, 1.63)* | *1.34 (1.19, 1.52)* | *1.26 (1.11, 1.42)* |
| **Both obesity and alcohol-related cancer (overlapping)** | | | | |
| **Never drinker** |  |  |  |  |
| Normal WC | 215 / 6,195 | *-* | *-* | *-* |
| Increased Risk WC | 172 / 4,070 | 1.17 (0.96, 1.43) | 1.17 (0.96, 1.43) | 1.14 (0.93, 1.39) |
| High Risk WC | 330 / 6,796 | *1.21 (1.02, 1.43)* | *1.20 (1.01, 1.43)* | 1.11 (0.93, 1.32) |
| **Previous drinker** |  |  |  |  |
| Normal WC | 191 / 5,040 | 1.20 (0.98, 1.46) | 1.13 (0.93, 1.38) | 1.13 (0.93, 1.38) |
| Increased Risk WC | 139 / 3,162 | *1.32 (1.07, 1.64)* | *1.25 (1.01, 1.55)* | 1.21 (0.98, 1.50) |
| High Risk WC | 289 / 5,446 | *1.49 (1.25, 1.78)* | *1.41 (1.18, 1.69)* | *1.31 (1.09, 1.56)* |
| **Within guideline** |  |  |  |  |
| Normal WC | 3,472 / 96,709 | 1.08 (0.94, 1.23) | 1.03 (0.90, 1.18) | 1.04 (0.91, 1.19) |
| Increased Risk WC | 2,443 / 56,605 | *1.20 (1.05, 1.38)* | *1.14 (0.99, 1.32)* | 1.12 (0.98, 1.29) |
| High Risk WC | 3,673 / 70,857 | *1.39 (1.21, 1.59)* | *1.31 (1.14, 1.51)* | *1.23 (1.08, 1.42)* |
| **Above guideline** |  |  |  |  |
| Normal WC | 1,953 / 61,888 | 1.13 (0.98, 1.31) | 1.05 (0.91, 1.21) | 1.06 (0.92, 1.22) |
| Increased Risk WC | 1,666 / 41,338 | *1.42 (1.23, 1.63)* | *1.30 (1.13, 1.50)* | *1.28 (1.11, 1.47)* |
| High Risk WC | 1,937 / 41,469 | *1.57 (1.36, 1.80)* | *1.43 (1.24, 1.65)* | *1.35 (1.17, 1.56)* |
| **Broad definition of alcohol-related cancer** | | | | |
| **Never drinker** |  |  |  |  |
| Normal WC | 284 / 6,195 | *-* | *-* | *-* |
| Increased Risk WC | 218 / 4,070 | 1.12 (0.94, 1.34) | 1.11 (0.93, 1.32) | 1.07 (0.9, 1.28) |
| High Risk WC | 420 / 6,796 | *1.18 (1.02, 1.37)* | 1.13 (0.98, 1.32) | 1.04 (0.89, 1.21) |
| **Previous drinker** |  |  |  |  |
| Normal WC | 296 / 5,040 | *1.36 (1.16, 1.60)* | *1.17 (1.00, 1.38)* | *1.17 (1.00, 1.37)* |
| Increased Risk WC | 229 / 3,162 | *1.58 (1.33, 1.88)* | *1.36 (1.14, 1.62)* | *1.31 (1.10, 1.55)* |
| High Risk WC | 441 / 5,446 | *1.69 (1.46, 1.96)* | *1.40 (1.21, 1.63)* | *1.29 (1.11, 1.50)* |
| **Within guideline** |  |  |  |  |
| Normal WC | 4,453 / 96,709 | 1.05 (0.93, 1.18) | 1.00 (0.88, 1.12) | 1.01 (0.90, 1.14) |
| Increased Risk WC | 3,169 / 56,605 | *1.17 (1.04, 1.33)* | 1.09 (0.96, 1.23) | 1.06 (0.94, 1.20) |
| High Risk WC | 4,740 / 70,857 | *1.36 (1.21, 1.54)* | *1.22 (1.08, 1.38)* | *1.15 (1.02, 1.29)* |
| **Above guideline** |  |  |  |  |
| Normal WC | 2,899 / 61,888 | *1.19 (1.05, 1.34)* | 1.05 (0.93, 1.18) | 1.06 (0.94, 1.20) |
| Increased Risk WC | 2,313 / 41,338 | *1.35 (1.19, 1.53)* | *1.17 (1.04, 1.33)* | *1.15 (1.01, 1.30)* |
| High Risk WC | 2,749 / 41,469 | *1.55 (1.37, 1.75)* | *1.32 (1.16, 1.49)* | *1.24 (1.09, 1.40)* |
| **Total cancer** | | | | |
| **Never drinker** |  |  |  |  |
| Normal WC | 747 / 6,195 | *-* | *-* | *-* |
| Increased Risk WC | 537 / 4,070 | 1.06 (0.94, 1.18) | 1.06 (0.95, 1.18) | 1.03 (0.92, 1.15) |
| High Risk WC | 945 / 6,796 | 1.08 (0.99, 1.19) | 1.09 (0.99, 1.20) | 1.02 (0.93, 1.13) |
| **Previous drinker** |  |  |  |  |
| Normal WC | 744 / 5,040 | *1.18 (1.07, 1.31)* | *1.14 (1.03, 1.26)* | *1.14 (1.03, 1.26)* |
| Increased Risk WC | 540 / 3,162 | *1.26 (1.13, 1.41)* | *1.23 (1.10, 1.37)* | *1.19 (1.06, 1.33)* |
| High Risk WC | 931 / 5,446 | *1.30 (1.18, 1.44)* | *1.27 (1.16, 1.40)* | *1.19 (1.08, 1.32)* |
| **Within guideline** |  |  |  |  |
| Normal WC | 13,163 / 96,709 | *1.17 (1.08, 1.26)* | *1.13 (1.05, 1.21)* | *1.14 (1.06, 1.23)* |
| Increased Risk WC | 8,706 / 56,605 | *1.20 (1.11, 1.29)* | *1.16 (1.07, 1.25)* | *1.14 (1.06, 1.23)* |
| High Risk WC | 11,493 / 70,857 | *1.28 (1.19, 1.38)* | *1.24 (1.15, 1.33)* | *1.18 (1.09, 1.27)* |
| **Above guideline** |  |  |  |  |
| Normal WC | 9,527 / 61,888 | *1.21 (1.12, 1.31)* | *1.14 (1.06, 1.23)* | *1.15 (1.07, 1.25)* |
| Increased Risk WC | 7,158 / 41,338 | *1.25 (1.16, 1.35)* | *1.18 (1.09, 1.27)* | *1.16 (1.07, 1.25)* |
| High Risk WC | 7,407 / 41,469 | *1.28 (1.19, 1.38)* | *1.21 (1.12, 1.31)* | *1.15 (1.07, 1.25)* |
| Cox proportional hazard model. Never drinker I Normal WC is the referent group.  Model 1 is adjusted for baseline age and sex. Model 2 is additionally adjusted for smoking status, dietary pattern score (determined by higher consumption of fruit, vegetables, and fish and lower consumption of processed meats and red meats (Rutten-Jacobs et al., 2018)), sleep duration (hrs/night), education, Townsend Deprivation Index and physical activity ((MET)-hour/week). Model 3 is further adjusted for chronic diseases (major cardiovascular disease (ICD-10 codes I00 to I99), Type 2 diabetes (ICD-10 codes E11.0 to E11.9 and E12) and dyslipidaemia (ICD-10 codes E78.0-E78.6) diagnosed by a doctor and hospital admission records and self-reported cardiovascular diseases and Type 2 diabetes).  Waist circumference was measured by using flexible plastic tape with the participant in the resting-standing position by a trained professional. WHO classification: normal (<80 cm for women, <94 cm for men), increased risk of metabolic complications (80-88 cm for women, 94-102 cm for men), substantially increased risk of metabolic complications (>88 cm for women, >102 cm for men).  Alcohol consumption categories are based on the average weekly intake of standard drinks relative to UK guidelines. In the UK, one standard drink equals to 8 g of pure alcohol. Within guidelines: ≤ 14 units/week; above guidelines:>14 units/week.  Alcohol-related cancer according to the broad definition included oral cavity, throat, larynx, esophagus, liver, colorectal, stomach, female breast, pancreas and lung cancer (IARC, 2014).  Alcohol-related cancer according to the narrow definition included oral cavity, throat, larynx, esophagus, liver, colorectal, stomach, female breast (IARC, 2014).  Obesity-related cancer included meningioma, multiple myeloma, adenocarcinoma of the esophagus, and cancers of the thyroid, postmenopausal breast, gallbladder, stomach, liver, pancreas, kidney, ovaries, uterus, colon and rectum (colorectal) (Lauby‑Secretan et al., 2016).  The definition of total cancer excludes in situ, benign, uncertain, or non-well-defined cancers.  *Italic font indicates statistical significance (p<0.05).* | | | | |

**Supplementary Table 8.** Joint association between alcohol consumption and BMI with cancer incidence by various definitions of cancer (N=399,575)

|  | Events / n | Model 1  HR (95% CI) | Model 2  HR (95% CI) | Model 3  HR (95% CI) |
| --- | --- | --- | --- | --- |
| **Narrow definition of alcohol-related cancer** | | | | |
| **Never drinker** |  |  |  |  |
| Normal weight | 207 / 5,080 | *-* | *-* | *-* |
| Overweight | 295 / 6,583 | 1.10 (0.92, 1.31) | 1.10 (0.92, 1.31) | 1.06 (0.89, 1.27) |
| Obese | 240 / 5,398 | 1.02 (0.85, 1.23) | 1.01 (0.84, 1.21) | 0.93 (0.77, 1.11) |
| **Previous drinker** |  |  |  |  |
| Normal weight | 201 / 3,799 | *1.31 (1.08, 1.59)* | *1.22 (1.00, 1.48)* | *1.22 (1.01, 1.48)* |
| Overweight | 251 / 4,950 | *1.31 (1.09, 1.57)* | *1.22 (1.01, 1.46)* | 1.17 (0.97, 1.41) |
| Obese | 239 / 4,447 | *1.38 (1.14, 1.66)* | *1.28 (1.06, 1.54)* | 1.17 (0.97, 1.41) |
| **Within guideline** |  |  |  |  |
| Normal weight | 3,243 / 77,738 | 1.04 (0.90, 1.19) | 0.99 (0.86, 1.14) | 1.01 (0.88, 1.16) |
| Overweight | 3,985 / 90,797 | 1.12 (0.98, 1.29) | 1.06 (0.92, 1.22) | 1.04 (0.91, 1.20) |
| Obese | 2,844 / 55,636 | *1.29 (1.12, 1.49)* | 1.22 (1.06, 1.40) | 1.13 (0.98, 1.31) |
| **Above guideline** |  |  |  |  |
| Normal weight | 1,808 / 43,466 | *1.16 (1.00, 1.34)* | 1.06 (0.92, 1.23) | 1.08 (0.93, 1.24) |
| Overweight | 2,784 / 67,836 | *1.30 (1.12, 1.49)* | *1.18 (1.03, 1.37)* | *1.16 (1.01, 1.41)* |
| Obese | 1,519 / 33,393 | *1.44 (1.25, 1.67)* | *1.31 (1.13, 1.51)* | *1.22 (1.05, 1.41)* |
| **Obesity-related cancer** | | | | |
| **Never drinker** |  |  |  |  |
| Normal weight | 249 / 5,080 | *-* | *-* | *-* |
| Overweight | 367 / 6,583 | 1.14 (0.97, 1.34) | 1.14 (0.97, 1.34) | 1.10 (0.93, 1.29) |
| Obese | 346 / 5,398 | *1.21 (1.03, 1.43)* | *1.22 (1.03, 1.43)* | 1.11 (0.94, 1.30) |
| **Previous drinker** |  |  |  |  |
| Normal weight | 204 / 4,000 | 1.12 (0.93, 1.35) | 1.07 (0.90, 1.29) | 1.07 (0.89, 1.29) |
| Overweight | 280 / 5,201 | *1.23 (1.04, 1.46)* | *1.19 (1.00, 1.41)* | 1.14 (0.96, 1.35) |
| Obese | 271 / 4,447 | *1.31 (1.10, 1.56)* | *1.27 (1.07, 1.51)* | 1.16 (0.97, 1.37) |
| **Within guideline** |  |  |  |  |
| Normal weight | 3,644 / 77,738 | 0.97 (0.86, 1.11) | 0.94 (0.82, 1.07) | 0.96 (0.84, 1.09) |
| Overweight | 4,636 / 90,797 | 1.09 (0.96, 1.24) | 1.05 (0.93, 1.20) | 1.03 (0.91, 1.17) |
| Obese | 3,552 / 55,636 | *1.35 (1.19, 1.54)* | *1.30 (1.14, 1.48)* | *1.20 (1.05, 1.37)* |
| **Above guideline** |  |  |  |  |
| Normal weight | 1,877 / 43,466 | 1.02 (0.89, 1.17) | 0.96 (0.84, 1.09) | 0.97 (0.85, 1.11) |
| Overweight | 3,027 / 67,836 | *1.22 (1.07, 1.39)* | *1.15 (1.00, 1.30)* | 1.12 (0.98, 1.27) |
| Obese | 1,671 / 33,393 | *1.38 (1.20, 1.57)* | *1.29 (1.13, 1.47)* | *1.19 (1.04, 1.36)* |
| **Obesity-related & narrow definition of alcohol-related cancer (combined)** | | | | |
| **Never drinker** |  |  |  |  |
| Normal weight | 265 / 5,080 | - | - | - |
| Overweight | 383 / 6,583 | 1.11 (0.95, 1.30) | 1.11 (0.95, 1.30) | 1.07 (0.91, 1.25) |
| Obese | 358 / 5,398 | *1.18 (1.01, 1.39)* | *1.18 (1.00, 1.38)* | 1.07 (0.91, 1.25) |
| **Previous drinker** |  |  |  |  |
| Normal weight | 230 / 4,000 | 1.17 (0.98, 1.40) | 1.11 (0.93, 1.32) | 1.11 (0.93, 1.33) |
| Overweight | 314 / 5,201 | *1.27 (1.08, 1.50)* | *1.21 (1.02, 1.42)* | 1.16 (0.98, 1.36) |
| Obese | 299 / 4,447 | *1.34 (1.14, 1.58)* | *1.27 (1.08, 1.50)* | 1.16 (0.98, 1.37) |
| **Within guideline** |  |  |  |  |
| Normal weight | 3,842 / 77,738 | 0.96 (0.85, 1.09) | 0.93 (0.82, 1.05) | 0.95 (0.84, 1.07) |
| Overweight | 4,909 / 90,797 | 1.08 (0.95, 1.22) | 1.04 (0.91, 1.17) | 1.01 (0.89, 1.15) |
| Obese | 3,715 / 55,636 | *1.32 (1.17, 1.49)* | *1.26 (1.11, 1.43)* | *1.17 (1.03, 1.32)* |
| **Above guideline** |  |  |  |  |
| Normal weight | 2,096 / 43,466 | 1.05 (0.92, 1.19) | 0.98 (0.86, 1.11) | 0.99 (0.87, 1.13) |
| Overweight | 3,320 / 67,836 | *1.20 (1.06, 1.36)* | 1.12 (0.99, 1.27) | 1.09 (0.96, 1.24) |
| Obese | 1,839 / 33,393 | *1.35 (1.19, 1.54)* | *1.26 (1.10, 1.43)* | *1.16 (1.02, 1.33)* |
| **Both obesity and alcohol-related cancer (overlapping)** | | | | |
| **Never drinker** |  |  |  |  |
| Normal weight | 195 / 5,080 | *-* | *-* | *-* |
| Overweight | 288 / 6,583 | 1.14 (0.95, 1.37) | 1.14 (0.95, 1.37) | 1.11 (0.92, 1.33) |
| Obese | 234 / 5,398 | 1.05 (0.87, 1.27) | 1.05 (0.86, 1.27) | 0.96 (0.79, 1.16) |
| **Previous drinker** |  |  |  |  |
| Normal weight | 176 / 4,000 | *1.23 (1.00, 1.51)* | 1.16 (0.95, 1.43) | 1.16 (0.95, 1.43) |
| Overweight | 224 / 5,201 | *1.27 (1.04, 1.53)* | 1.20 (0.97, 1.45) | 1.15 (0.95, 1.40) |
| Obese | 219 / 4,447 | *1.36 (1.12, 1.65)* | *1.29 (1.06, 1.56)* | 1.18 (0.97, 1.43) |
| **Within guideline** |  |  |  |  |
| Normal weight | 3,087 / 77,738 | 1.05 (0.91, 1.21) | 1.00 (0.87, 1.16) | 1.02 (0.88, 1.18) |
| Overweight | 3,780 / 90,797 | 1.14 (0.99, 1.32) | 1.09 (0.94, 1.25) | 1.06 (0.92, 1.23) |
| Obese | 2,721 / 55,636 | *1.32 (1.14, 1.53)* | *1.25 (1.08, 1.45)* | *1.17 (1.01, 1.35)* |
| **Above guideline** |  |  |  |  |
| Normal weight | 1,613 / 43,466 | 1.12 (0.97, 1.30) | 1.04 (0.89, 1.20) | 1.05 (0.90, 1.22) |
| Overweight | 2,562 / 67,836 | *1.33 (1.15, 1.54)* | *1.22 (1.06, 1.42)* | *1.20 (1.03, 1.39)* |
| Obese | 1,381 / 33,393 | *1.46 (1.26, 1.70)* | *1.34 (1.15, 1.56)* | *1.25 (1.07, 1.45)* |
| **Broad definition of alcohol-related cancer** | | | | |
| **Never drinker** |  |  |  |  |
| Normal weight | 261 / 5,080 | *-* | *-* | *-* |
| Overweight | 358 / 6,583 | 1.04 (0.89, 1.22) | 1.03 (0.88, 1.21) | 0.99 (0.85, 1.17) |
| Obese | 303 / 5,095 | 1.01 (0.86, 1.20) | 0.98 (0.83, 1.16) | 0.90 (0.76, 1.06) |
| **Previous drinker** |  |  |  |  |
| Normal weight | 269 / 3,731 | *1.37 (1.16, 1.63)* | *1.18 (0.99, 1.40)* | *1.18 (1.00, 1.40)* |
| Overweight | 372 / 5,201 | *1.47 (1.25, 1.72)* | *1.27 (1.08, 1.48)* | *1.21 (1.04, 1.42)* |
| Obese | 325 / 4,447 | *1.44 (1.23, 1.72)* | *1.21 (1.03, 1.42)* | 1.10 (0.94, 1.30) |
| **Within guideline** |  |  |  |  |
| Normal weight | 3,904 / 77,738 | 1.00 (0.88, 1.14) | 0.96 (0.84, 1.08) | 0.97 (0.86, 1.10) |
| Overweight | 4,940 / 90,797 | 1.08 (0.95, 1.22) | 1.00 (0.89, 1.14) | 0.98 (0.87, 1.11) |
| Obese | 3,518 / 55,636 | *1.26 (1.11, 1.43)* | 1.14 (1.00, 1.29) | 1.05 (0.93, 1.19) |
| **Above guideline** |  |  |  |  |
| Normal weight | 2,365 / 43,466 | *1.17 (1.03, 1.34)* | 1.03 (0.91, 1.17) | 1.05 (0.92, 1.19) |
| Overweight | 3,645 / 67,836 | *1.25 (1.10, 1.41)* | 1.09 (0.96, 1.24) | 1.07 (0.94, 1.21) |
| Obese | 1,951 / 33,393 | *1.36 (1.19, 1.55)* | *1.17 (1.03, 1.33)* | 1.08 (0.95, 1.23) |
| **Total cancer** | | | | |
| **Never drinker** |  |  |  |  |
| Normal weight | 637 / 5,080 | *-* | *-* | *-* |
| Overweight | 868 / 6,583 | 0.98 (0.89, 1.09) | 0.99 (0.89, 1.10) | 0.96 (0.86, 1.06) |
| Obese | 724 / 5,398 | 1.01 (0.91, 1.12) | 1.02 (0.92, 1.14) | 0.95 (0.85, 1.06) |
| **Previous drinker** |  |  |  |  |
| Normal weight | 609 / 4,000 | *1.18 (1.06, 1.32)* | *1.14 (1.02, 1.27)* | *1.14 (1.02, 1.27)* |
| Overweight | 879 / 5,201 | *1.20 (1.08, 1.33)* | *1.16 (1.05, 1.29)* | *1.13 (1.02, 1.25)* |
| Obese | 727 / 4,447 | *1.19 (1.07, 1.32)* | *1.17 (1.05, 1.30)* | 1.08 (0.97, 1.21) |
| **Within guideline** |  |  |  |  |
| Normal weight | 10,655 / 77,738 | *1.13 (1.04, 1.22)* | *1.09 (1.01, 1.18)* | *1.11 (1.02, 1.20)* |
| Overweight | 13,974 / 90,797 | *1.14 (1.06, 1.24)* | *1.11 (1.02, 1.20)* | *1.09 (1.00, 1.18)* |
| Obese | 8,733 / 55,636 | *1.21 (1.12, 1.31)* | *1.18 (1.08, 1.27)* | *1.11 (1.02, 1.20)* |
| **Above guideline** |  |  |  |  |
| Normal weight | 6,691 / 43,466 | *1.17 (1.08, 1.27)* | *1.11 (1.02, 1.20)* | *1.12 (1.03, 1.21)* |
| Overweight | 11,763 / 67,836 | *1.20 (1.11, 1.30)* | *1.14 (1.05, 1.23)* | *1.11 (1.03, 1.21)* |
| Obese | 5,638 / 33,393 | *1.18 (1.08, 1.28)* | *1.12 (1.03, 1.21)* | 1.05 (0.97, 1.14) |
| Cox proportional hazard model. Never drinker I Normal weight is the referent group.  Model 1 is adjusted for baseline age and sex. Model 2 is additionally adjusted for smoking status, dietary pattern score (determined by higher consumption of fruit, vegetables, and fish and lower consumption of processed meats and red meats (Rutten-Jacobs et al., 2018)), sleep duration (hrs/night), education, Townsend Deprivation Index and physical activity ((MET)-hour/week). Model 3 is further adjusted for chronic diseases (major cardiovascular disease (ICD-10 codes I00 to I99), Type 2 diabetes (ICD-10 codes E11.0 to E11.9 and E12) and dyslipidaemia (ICD-10 codes E78.0-E78.6) diagnosed by a doctor and hospital admission records and self-reported cardiovascular diseases and Type 2 diabetes). Body mass index = Weight (kg)/height (m^2^). WHO classification: normal weight (<25 kg/m^2^), overweight and obese (≥ 25 kg/m^2^).  Alcohol consumption categories are based on the average weekly intake of standard drinks relative to UK guidelines. In the UK, one standard drink equals to 8 g of pure alcohol. Within guidelines: ≤ 14 units/week; above guidelines:>14 units/week.  Alcohol-related cancer according to the broad definition included oral cavity, throat, larynx, esophagus, liver, colorectal, stomach, female breast, pancreas and lung cancer (IARC, 2014).  Alcohol-related cancer according to the narrow definition included oral cavity, throat, larynx, esophagus, liver, colorectal, stomach, female breast (IARC, 2014).  Obesity-related cancer included meningioma, multiple myeloma, adenocarcinoma of the esophagus, and cancers of the thyroid, postmenopausal breast, gallbladder, stomach, liver, pancreas, kidney, ovaries, uterus, colon and rectum (colorectal) (Lauby‑Secretan et al., 2016).  The definition of total cancer excludes in situ, benign, uncertain, or non-well-defined cancers.  *Italic font indicates statistical significance (p<0.05).* | | | | |

**Supplementary Table 9.** Joint association between alcohol consumption and adiposity markers with cancer incidence by various definitions of cancer with exclusion of events occurring in the first 3 years of follow-up

|  | **Narrow definition of alcohol-related cancer** | | **Obesity-related cancer** | |
| --- | --- | --- | --- | --- |
|  | Events / n | HR (95% CI) | Events / n | HR (95% CI) |
| **Joint group of alcohol consumption and BF%** | 13,151/385,110 |  | 15,115 / 394,566 |  |
| **Never drinker** |  |  |  |  |
| Bottom Tertile BF% | 81 / 4,670 | *-* | 104 / 4,663 | *-* |
| Middle Tertile BF% | 35 / 997 | 1.47 (0.98, 2.20) | 40 / 995 | 1.28 (0.88, 1.85) |
| Top Tertile BF% | 448 / 11,216 | *1.33 (1.03, 1.71)* | 588 / 11,173 | *1.31 (1.04, 1.64)* |
| **Previous drinker** |  |  |  |  |
| Bottom Tertile BF% | 151 / 5,534 | *1.40 (1.07, 1.83)* | 153 / 5,535 | 1.14 (0.88, 1.46) |
| Middle Tertile BF% | 37 / 1,028 | *1.48 (1.00, 2.20)* | 39 / 1,030 | 1.22 (0.84, 1.77) |
| Top Tertile BF% | 322 / 6,905 | *1.45 (1.12, 1.89)* | 372 / 6,892 | *1.29 (1.02, 1.63)* |
| **Within guideline** |  |  |  |  |
| Bottom Tertile BF% | 1,471 / 74,226 | 1.10 (0.88, 1.38) | 1,650 / 74,206 | 0.97 (0.80, 1.18) |
| Middle Tertile BF% | 502 / 15,221 | *1.41 (1.11, 1.80)* | 563 / 15,212 | 1.23 (0.99, 1.53) |
| Top Tertile BF% | 5,469 / 132,094 | *1.41 (1.11, 1.80)* | 6,598 / 131,732 | *1.30 (1.05, 1.61)* |
| **Above guideline** |  |  |  |  |
| Bottom Tertile BF% | 2,188 / 87,988 | *1.32 (1.06, 1.65)* | 2,249 / 87,996 | 1.08 (0.89, 1.32) |
| Middle Tertile BF% | 359 / 9,220 | *1.65 (1.29, 2.10)* | 362 / 9,219 | *1.30 (1.04, 1.63)* |
| Top Tertile BF% | 2,088 / 46,011 | *1.55 (1.22, 1.97)* | 2,397 / 45,913 | *1.38 (1.11, 1.71)* |
| **Joint group of alcohol consumption and WC** | 13,151/385,110 |  | 15,115 / 394566 |  |
| **Never drinker** |  |  |  |  |
| Normal WC | 167 / 6,135 | *-* | 204 / 6,125 | *-* |
| Increased Risk WC | 135 / 4,028 | 1.11 (0.88, 1.39) | 175 / 4,019 | 1.18 (0.96, 1.44) |
| High Risk WC | 262 / 6,720 | 1.10 (0.90, 1.33) | 353 / 6,687 | *1.21 (1.01, 1.43)* |
| **Previous drinker** |  |  |  |  |
| Normal WC | 168 / 4,987 | *1.25 (1.01, 1.55)* | 168 / 4,984 | 1.07 (0.87, 1.31) |
| Increased Risk WC | 111 / 3,115 | 1.19 (0.93, 1.51) | 126 / 3,116 | 1.14 (0.92, 1.43) |
| High Risk WC | 231 / 5,365 | *1.25 (1.03, 1.53)* | 270 / 5,357 | *1.24 (1.03, 1.49)* |
| **Within guideline** |  |  |  |  |
| Normal WC | 2,677 / 95,699 | 1.05 (0.90, 1.23) | 3,019 / 95,583 | 0.99 (0.85, 1.13) |
| Increased Risk WC | 1,889 / 55,939 | 1.12 (0.96, 1.32) | 2,205 / 55,838 | 1.09 (0.94, 1.26) |
| High Risk WC | 2,876 / 69,903 | *1.22 (1.04, 2.42)* | 3,587 / 69,729 | *1.26 (1.10, 1.45)* |
| **Above guideline** |  |  |  |  |
| Normal WC | 1,643 / 61,324 | 1.14 (0.97, 1.34) | 1,737 / 61,301 | 1.04 (0.90, 1.20) |
| Increased Risk WC | 1,380 / 40,924 | *1.33 (1.13, 1.57)* | 1,499 / 40,904 | *1.25 (1.08, 1.45)* |
| High Risk WC | 1,612 / 40,971 | *1.40 (1.19, 1.64)* | 1,772 / 40,923 | *1.32 (1.14, 1.53)* |
| **Joint group of alcohol consumption and BMI** | 13,151/385,110 |  | 15,115 / 394566 |  |
| **Never drinker** |  |  |  |  |
| Normal weight | 154 / 5,027 | *-* | 185 / 5,016 | - |
| Overweight | 228 / 6,516 | 1.08 (0.88, 1.33) | 287 / 6,503 | 1.14 (0.95, 1.37) |
| Obese | 182 / 5,340 | 0.93 (0.75, 1.15) | 260 / 5,312 | 1.12 (0.92, 1.35) |
| **Previous drinker** |  |  |  |  |
| Normal weight | 154 / 3,953 | *1.27 (1.02, 1.59)* | 153 / 3,949 | 1.09 (0.88, 1.35) |
| Overweight | 174 / 5,124 | 1.08 (0.87, 1.34) | 199 / 5,120 | 1.07 (0.88, 1.31) |
| Obese | 182 / 4,390 | 1.18 (0.95, 1.46) | 212 / 4,388 | 1.19 (0.98, 1.45) |
| **Within guideline** |  |  |  |  |
| Normal weight | 2,361 / 76,856 | 1.03 (0.87, 1.21) | 2,657 / 76,751 | 0.97 (0.83, 1.13) |
| Overweight | 2,929 / 89,741 | 1.05 (0.89, 1.23) | 3,427 / 89,588 | 1.04 (0.90, 1.21) |
| Obese | 2,152 / 54,944 | 1.14 (0.97, 1.35) | 2,727 / 54,811 | *1.24 (1.07, 1.44)* |
| **Above guideline** |  |  |  |  |
| Normal weight | 1,337 / 42,995 | 1.23 (0.96, 1.34) | 1,391 / 42,980 | 1.02 (0.88, 1.19) |
| Overweight | 2,122 / 67,174 | *1.23 (1.04, 1.45)* | 2,322 / 67,131 | *1.19 (1.02, 1.39)* |
| Obese | 1,176 / 33,050 | *1.30 (1.09, 2.54)* | 1,295 / 33,017 | *1.27 (1.09, 1.49)* |
| Cox proportional hazard model.  Model is adjusted for baseline age, sex, smoking status, dietary pattern score (determined by higher consumption of fruit, vegetables, and fish and lower consumption of processed meats and red meats (Rutten-Jacobs et al., 2018)), sleep duration (hrs/night), education, Townsend Deprivation Index and physical activity ((MET)-hour/week), chronic diseases (major cardiovascular disease (ICD-10 codes I00 to I99), Type 2 diabetes (ICD-10 codes E11.0 to E11.9 and E12) and dyslipidaemia (ICD-10 codes E78.0-E78.6) diagnosed by a doctor and hospital admission records and self-reported cardiovascular diseases and Type 2 diabetes).  Body Fat (BF%) was measured by bioimpedance using the Tanita BC-418MA device (Tanita, Tokyo, Japan). BF% by tertile: Bottom Tertile 1: <23.1% for women and <33.9% for men, Middle Tertile: 23.1-27.8% for women and 33.9-39.8 for men, Top Tertile: >27.8% for women and >39.8% for men. Waist circumference was measured by using flexible plastic tape with the participant in the resting-standing position by a trained professional. WHO classification: normal (<80 cm for women, <94 cm for men), increased risk of metabolic complications (80-88 cm for women, 94-102 cm for men), substantially increased risk of metabolic complications (>88 cm for women, >102 cm for men). Body mass index = Weight (kg)/height (m^2^). WHO classification: normal weight (<25 kg/m^2^), overweight and obese (≥ 25 kg/m^2^).  Alcohol consumption categories are based on the average weekly intake of standard drinks relative to UK guidelines. In the UK, one standard drink equals to 8 g of pure alcohol. Within guidelines: ≤ 14 units/week; above guidelines:>14 units/week.  Alcohol-related cancer according to the narrow definition included oral cavity, throat, larynx, esophagus, liver, colorectal, stomach, female breast (IARC, 2014).  Obesity-related cancer included meningioma, multiple myeloma, adenocarcinoma of the esophagus, and cancers of the thyroid, postmenopausal breast, gallbladder, stomach, liver, pancreas, kidney, ovaries, uterus, colon and rectum (colorectal) (Lauby‑Secretan et al., 2016).  *Italic font indicates statistical significance (p<0.05).* | | | | |

**Supplementary Table 10.** Joint association between alcohol consumption and adiposity markers with incidence of different cancer-sites (N=399,575)

|  | **Oral cavity cancer** | | **Throat cancer** | | **Oesophagus cancer** | | **Liver cancer** | | **Colorectal cancer** | | **Stomach cancer** | | **Breast cancer** | |
| --- | --- | --- | --- | --- | --- | --- | --- | --- | --- | --- | --- | --- | --- | --- |
|  | Events / n | HR (95% CI) | Events / n | HR (95% CI) | Events / n | HR (95% CI) | Events / n | HR (95% CI) | Events / n | HR (95% CI) | Events / n | HR (95% CI) | Events / n | HR (95% CI) |
| **Joint group of alcohol consumption and BF%** | | | | | | | | | | | | | | |
| **Never drinker** | | | | | | | | | | | | | | |
| Bottom Tertile BF% | 6 /3951 | - | 3 /3951 | - | 8 /3951 | - | 5 /3951 |  | 41 /3951 |  | 8 /3951 |  | 24 /3951 |  |
| Middle Tertile BF% | 8 /5073 | 3.38 (0.79, 14.37) | 2 /5073 | 4.46 (0.73, 27.06) | 17 /5073 | *3.97 (1.55, 10.14)* | 13 /5073 | 0.55 (0.07, 4.18) | 63 /5073 | 0.85 (0.42, 1.72) | 16 /5073 | 1.75 (0.57, 5.31) | 111 /5073 | 1.98 (0.75, 5.23) |
| Top Tertile BF% | 5 / 8585 | 1.35 (0.43, 4.22) | 3 / 8585 | 0.87 (0.16, 4.65) | 16 / 8585 | 1.81 (0.82, 3.98) | 21 / 8585 | *2.32 (1.04, 5.14)* | 100 / 8585 | 1.20 (0.84, 1.70) | 17 / 8585 | 1.08 (0.49, 2.40) | 312 / 8585 | 1.96 (0.81, 4.74) |
| **Previous drinker** | | | | | | | | | | | | | | |
| Bottom Tertile BF% | 16 / 4153 | *3.53 (1.28, 8.78)* | 15 / 4153 | *4.66 (1.40, 15.51)* | 25 / 4153 | *2.03 (1.06, 3.88)* | 13 / 4153 | 1.17 (0.59, 2.30) | 55 / 4153 | 1.07 (0.75, 1.50) | 11 / 4153 | 1.10 (0.58, 2.09) | 21 / 4153 | 2.22 (0.76, 6.49) |
| Middle Tertile BF% | 16 / 4338 | 2.50 (0.59, 10.54) | 14 / 4338 | 1.30 (0.14, 12.60) | 26 / 4338 | 2.05 (0.77, 5.48) | 22 / 4338 | 1.28 (0.42, 3.95) | 67 / 4338 | 1.39 (0.81, 2.37) | 25 / 4338 | *3.12 (1.43, 6.84)* | 78 / 4338 | 1.38 (0.49, 3.92) |
| Top Tertile BF% | 12 / 5348 | 2.58 (0.87, 7.65) | 5 / 5348 | 2.73 (0.67, 11.10) | 12 / 5348 | 1.84 (0.81, 4.16) | 14 / 5348 | *2.61 (1.16, 5.86)* | 74 / 5348 | 1.30 (0.90, 1.87) | 14 / 5348 | 1.14 (0.50, 2.63) | 198 / 5348 | 2.15 (0.89, 5.22) |
| **Within guideline** | | | | | | | | | | | | | | |
| Bottom Tertile BF% | 104 / 63485 | 1.51 (0.62, 3.70) | 74 / 63485 | 1.91 (0.60, 6.04) | 161 / 63485 | 1.24 (0.69, 2.21) | 94 / 63485 | 0.71 (0.40, 1.25) | 792 / 63485 | 1.14 (0.87, 1.49) | 150 / 63485 | 0.87 (0.51, 1.47) | 438 / 63485 | 1.71 (0.70, 4.18) |
| Middle Tertile BF% | 105 / 73687 | 1.84 (0.69, 4.94) | 48 / 73687 | 2.08 (0.59, 7.34) | 179 / 73687 | *1.90 (1.01, 3.60)* | 137 / 73687 | 1.05 (0.54, 2.05) | 909 / 73687 | 1.20 (0.88, 1.64) | 148 / 73687 | 0.88 (0.47, 1.67) | 1847 / 73687 | 2.04 (0.84, 4.94) |
| Top Tertile BF% | 124 / 89167 | 1.73 (0.65, 4.62) | 53 / 89167 | 1.50 (0.42, 5.33) | 148 / 89167 | 1.21 (0.61, 2.39) | 152 / 89167 | 1.76 (0.87, 3.56) | 1126 / 89167 | 1.19 (0.87, 1.62) | 135 / 89167 | 0.95 (0.49, 1.86) | 3594 / 89167 | 2.24 (0.93, 5.39) |
| **Above guideline** | | | | | | | | | | | | | | |
| Bottom Tertile BF% | 147 / 64273 | 1.86 (0.77, 4.54) | 134 / 64273 | 2.60 (0.83, 8.17) | 226 / 64273 | 1.32 (0.74, 2.35) | 99 / 64273 | 0.72 (0.41, 1.26) | 1003 / 64273 | *1.37 (1.05, 1.80)* | 179 / 64273 | 0.88 (0.52, 1.49) | 201 / 64273 | 2.11 (0.85, 5.22) |
| Middle Tertile BF% | 116 / 50718 | 2.02 (0.76, 5.40) | 72 / 50718 | 3.64 (1.08, 12.26) | 217 / 50718 | 1.79 (0.95, 3.39) | 150 / 50718 | 1.41 (0.75, 2.65) | 911 / 50718 | 1.35 (0.99, 1.83) | 141 / 50718 | 0.71 (0.37, 1.38) | 800 / 50718 | 2.12 (0.87, 5.17) |
| Top Tertile BF% | 39 / 30606 | 1.59 (0.59, 4.33) | 26 / 30606 | 1.33 (0.36, 4.88) | 61 / 30606 | 1.48 (0.73, 2.99) | 57 / 30606 | 1.36 (0.66, 2.82) | 437 / 30606 | 1.25 (0.91, 1.72) | 41 / 30606 | 0.80 (0.39, 1.60) | 1314 / 30606 | *2.48 (1.03, 5.97)* |
| **Joint group of alcohol consumption and WC** | | | | | | | | | | | | | | |
| **Never drinker** | | | | | | | | | | | | | | |
| Normal WC | 12 / 6358 | *-* | 2 / 6358 | *-* | 14 / 6358 | *-* | 7 / 6358 |  | 75 / 6358 |  | 10 / 6358 |  | 119 / 6358 |  |
| Increased Risk WC | 2 / 4206 | 0.24 (0.06, 1.34) | 3 / 4206 | 2.30 (0.38, 13.77) | 7 / 4206 | 0.69 (0.28, 1.70) | 10 / 4206 | 1.68 (0.62, 4.50) | 45 / 4206 | 0.79 (0.54, 1.15) | 8 / 4206 | 1.21 (0.47, 3.14) | 115 / 4206 | *1.34 (1.03, 1.74)* |
| High Risk WC | 5 / 7045 | *0.35 (1.12, 1.43)* | 3 / 7045 | 1.38 (0.23, 8.28) | 20 / 7045 | 1.15 (0.58, 2.27) | 22 / 7045 | 2.15 (0.92, 5.04) | 84 / 7045 | 0.86 (0.63, 1.18) | 23 / 7045 | 1.84 (0.84, 4.04) | 203 / 7045 | 1.17 (0.93, 1.47) |
| **Previous drinker** | | | | | | | | | | | | | | |
| Normal WC | 17 / 5102 | 1.32 (0.63, 1.35) | 15 / 5102 | *5.53 (1.26, 24.22)* | 19 / 5102 | 1.14 (0.57, 2.27) | 15 / 5102 | 2.01 (0.82, 4.93) | 70 / 5102 | 1.02 (0.73, 1.42) | 11 / 5102 | 1.13 (0.47, 2.74) | 86 / 5102 | 1.11 (0.84, 1.47) |
| Increased Risk WC | 10 / 3208 | 1.15 (0.49, 1.46) | 9 / 3208 | *4.86 (1.05, 22.56)* | 20 / 3208 | 1.59 (0.80, 3.16) | 10 / 3208 | 1.71 (0.65, 4.50) | 37 / 3208 | 0.75 (0.50, 1.11) | 14 / 3208 | 1.94 (0.84, 4.49) | 66 / 3208 | 1.32 (0.98, 1.78) |
| High Risk WC | 17 / 5529 | 1.12 (0.53, 1.56) | 10 / 5529 | 3.14 (0.69, 14.40) | 24 / 5529 | 1.11 (0.57, 2.16) | 24 / 5529 | 2.11 (0.90, 4.93) | 89 / 5529 | 1.03 (0.76, 1.41) | 25 / 5529 | 1.97 (0.92, 4.25) | 145 / 5529 | *1.33 (1.04, 1.71)* |
| **Within guideline** | | | | | | | | | | | | | | |
| Normal WC | 146 / 97468 | 0.74 (0.41, 1.11) | 84 / 97468 | 2.69 (0.66, 10.95) | 135 / 97468 | 0.58 (0.34, 1.01) | 125 / 97468 | 1.18 (0.55, 2.52) | 1028 / 97468 | 0.88 (0.69, 1.11) | 146 / 97468 | 1.04 (0.53, 2.04) | 2169 / 97468 | *1.21 (1.01, 1.46)* |
| Increased Risk WC | 79 / 57202 | 0.61 (0.33, 1.24) | 35 / 57202 | 1.69 (0.41, 7.04) | 138 / 57202 | 0.83 (0.48, 1.45) | 80 / 57202 | 1.00 (0.46, 2.18) | 738 / 57202 | 0.92 (0.72, 1.16) | 110 / 57202 | 1.10 (0.56, 2.18) | 1485 / 57202 | *1.35 (1.11, 1.63)* |
| High Risk WC | 108 / 71669 | 0.63 (0.35, 1.54) | 56 / 71669 | 1.95 (1.47, 8.01) | 215 / 71669 | 0.97 (0.57, 1.68) | 178 / 71669 | 1.57 (0.73, 3.34) | 1061 / 71669 | 1.00 (0.79, 1.27) | 177 / 71669 | 1.33 (0.68, 2.61) | 2225 / 71669 | *1.40 (1.16, 1.69)* |
| **Above guideline** | | | | | | | | | | | | | | |
| Normal WC | 130 / 62222 | 0.79 (0.44, 1.17) | 120 / 62222 | 3.59 (0.89, 14.59) | 165 / 62222 | 0.76 (0.44, 1.32) | 74 / 62222 | 0.83 (0.38, 1.80) | 788 / 62222 | 0.94 (0.74, 1.19) | 104 / 62222 | 0.86 (0.43, 1.71) | 899 / 62222 | *1.34 (1.10, 1.63)* |
| Increased Risk WC | 74 / 41603 | 0.62 (0.33, 1.39) | 47 / 41603 | 1.91 (0.46, 7.89) | 159 / 41603 | 0.90 (0.52, 1.55) | 82 / 41603 | 1.10 (0.51, 2.39) | 762 / 41603 | 1.17 (0.92, 1.48) | 128 / 41603 | 1.29 (0.66, 2.56) | 625 / 41603 | *1.47 (1.21, 1.80)* |
| High Risk WC | 89 / 41772 | 0.78 (0.43, 1.57) | 65 / 41772 | 2.48 (0.61, 10.19) | 180 / 41772 | 0.93 (0.54, 1.62) | 150 / 41772 | 1.72 (0.80, 3.70) | 801 / 41772 | 1.14 (0.90, 1.45) | 129 / 41772 | 1.21 (0.61, 2.39) | 791 / 41772 | *1.57 (1.29, 1.92)* |
| **Joint group of alcohol consumption and BMI** | | | | | | | | | | | | | | |
| **Never drinker** | | | | | | | | | | | | | | |
| Normal weight | 12 / 5207 | - | 2 / 5207 | - | 12 / 5207 | - | 7 / 5207 |  | 61 / 5207 |  | 9 / 5207 |  | 119 / 5207 |  |
| Overweight | 4 / 6818 | *0.24 (0.08, 0.73)* | 3 / 6818 | 1.05 (0.18, 6.28) | 12 / 6818 | 0.64 (0.29, 1.43) | 16 / 6818 | 1.35 (0.55, 3.30) | 89 / 6818 | 0.98 (0.70, 1.36) | 11 / 6818 | 0.81 (0.32, 2.04) | 174 / 6818 | *1.18 (0.93, 1.50)* |
| Obese | 3 / 5584 | *0.20 (0.06, 0.73)* | 3 / 5584 | 1.25 (0.21, 7.48) | 17 / 5584 | 1.11 (0.53, 2.32) | 16 / 5584 | 1.57 (0.65, 3.83) | 54 / 5584 | 0.72 (0.50, 1.03) | 21 / 5584 | 1.93 (0.85, 4.40) | 144 / 5584 | 1.04 (0.81, 1.33) |
| **Previous drinker** | | | | | | | | | | | | | | |
| Normal weight | 12 / 4043 | 0.97 (0.43, 2.16) | 15 / 4043 | *5.72 (1.30, 25.05)* | 16 / 4043 | 1.18 (0.56, 2.49) | 11 / 4043 | 1.56 (0.60, 4.02) | 54 / 4043 | 1.01 (0.69, 1.46) | 6 / 4043 | 0.74 (0.26, 2.13) | 96 / 4043 | 1.23 (0.94, 1.61) |
| Overweight | 16 / 5289 | 0.88 (0.42, 1.87) | 13 / 5289 | 3.20 (0.72, 14.22) | 27 / 5289 | 1.18 (0.60, 2.34) | 20 / 5289 | 1.58 (0.66, 3.77) | 74 / 5289 | 0.92 (0.66, 1.30) | 21 / 5289 | 1.54 (0.68, 3.49) | 98 / 5289 | 1.12 (0.85, 1.47) |
| Obese | 16 / 4507 | 0.99 (0.47, 2.11) | 6 / 4507 | 1.65 (0.33, 8.21) | 20 / 4507 | 1.03 (0.50, 2.12) | 18 / 4507 | 1.62 (0.67, 3.90) | 68 / 4507 | 1.00 (0.70, 1.40) | 23 / 4507 | 1.96 (0.87, 4.39) | 103 / 4507 | 1.18 (0.90, 1.54) |
| **Within guideline** | | | | | | | | | | | | | | |
| Normal weight | 114 / 78320 | 0.60 (0.33, 1.08) | 58 / 78320 | 1.94 (0.47, 7.95) | 91 / 78320 | 0.50 (0.27, 0.91) | 93 / 78320 | 0.94 (0.43, 2.02) | 838 / 78320 | 0.92 (0.71, 1.19) | 101 / 78320 | 0.87 (0.42, 1.79) | 2066 / 78320 | 1.17 (0.97, 1.41) |
| Overweight | 143 / 91718 | *0.54 (0.30, 0.98)* | 75 / 91718 | 1.66 (0.41, 6.77) | 223 / 91718 | 0.74 (0.41, 1.33) | 142 / 91718 | 0.88 (0.41, 1.88) | 1190 / 91718 | 0.93 (0.72, 1.20) | 84 / 91718 | 0.99 (0.48, 2.00) | 2209 / 91718 | *1.24 (1.03, 1.50)* |
| Obese | 76 / 56301 | *0.45 (0.24, 0.82)* | 42 / 56301 | 1.31 (0.32, 5.45) | 174 / 56301 | 0.89 (0.50, 1.61) | 148 / 56301 | 1.32 (0.61, 2.82) | 799 / 56301 | 0.98 (0.76, 1.28) | 148 / 56301 | 1.22 (0.60, 2.49) | 1604 / 56301 | *1.31 (1.08, 1.58)* |
| **Above guideline** | | | | | | | | | | | | | | |
| Normal weight | 107 / 43665 | 0.77 (0.42, 1.41) | 86 / 43665 | 2.99 (0.73, 12.21) | 104 / 43665 | 0.70 (0.38, 1.28) | 59 / 43665 | 0.81 (0.37, 1.79) | 529 / 43665 | 0.93 (0.71, 1.22) | 66 / 43665 | 0.77 (0.37, 1.60) | 910 / 43665 | *1.29 (1.06, 1.57)* |
| Overweight | 123 / 68273 | *0.48 (0.26, 0.88)* | 93 / 68273 | 1.70 (0.42, 6.93) | 251 / 68273 | 0.79 (0.44, 1.42) | 124 / 68273 | 0.83 (0.38, 1.78) | 1203 / 68273 | 1.15 (0.88, 1.49) | 195 / 68273 | 1.07 (0.52, 2.18) | 929 / 68273 | *1.41 (1.16, 1.71)* |
| Obese | 72 / 33659 | 0.55 (0.30, 1.03) | 53 / 33659 | 1.78 (0.43, 7.35) | 149 / 33659 | 0.86 (0.48, 1.57) | 123 / 33659 | 1.40 (0.65, 3.02) | 619 / 33659 | 1.12 (0.86, 1.46) | 100 / 33659 | 1.01 (0.49, 2.09) | 476 / 33659 | *1.42 (1.16, 1.74)* |
| Cox proportional hazard model.  Model is adjusted for baseline age, sex, smoking status, dietary pattern score (determined by higher consumption of fruit, vegetables, and fish and lower consumption of processed meats and red meats (Rutten-Jacobs et al., 2018)), sleep duration (hrs/night), education, Townsend Deprivation Index and physical activity ((MET)-hour/week), chronic diseases (major cardiovascular disease (ICD-10 codes I00 to I99), Type 2 diabetes (ICD-10 codes E11.0 to E11.9 and E12) and dyslipidaemia (ICD-10 codes E78.0-E78.6) diagnosed by a doctor and hospital admission records and self-reported cardiovascular diseases and Type 2 diabetes).  Body Fat (BF%) was measured by bioimpedance using the Tanita BC-418MA device (Tanita, Tokyo, Japan). BF% by tertile: Bottom Tertile 1: <23.1% for women and <33.9% for men, Middle Tertile: 23.1-27.8% for women and 33.9-39.8 for men, Top Tertile: >27.8% for women and >39.8% for men. Waist circumference was measured by using flexible plastic tape with the participant in the resting-standing position by a trained professional. WHO classification: normal (<80 cm for women, <94 cm for men), increased risk of metabolic complications (80-88 cm for women, 94-102 cm for men), substantially increased risk of metabolic complications (>88 cm for women, >102 cm for men). Body mass index = Weight (kg)/height (m^2^). WHO classification: normal weight (<25 kg/m^2^), overweight and obese (≥ 25 kg/m^2^).  Alcohol consumption categories are based on the average weekly intake of standard drinks relative to UK guidelines. In the UK, one standard drink equals to 8 g of pure alcohol. Within guidelines: ≤ 14 units/week; above guidelines:>14 units/week.  *Italic font indicates statistical significance (p<0.05).* | | | | | | | | | | | | | | |

**Supplementary Table 11.** Three-way Interaction between alcohol consumption, adiposity markers and smoking for cancer incidence by various definitions of cancer (N=385,927)*

|  | Alcohol consumption*BF%*smoking | Alcohol consumption*WC*smoking | Alcohol consumption*BMI*smoking |
| --- | --- | --- | --- |
|  | *P* | *P* | *P* |
| **Narrow definition of alcohol-related cancer** | 0.003 | <0.001 | <0.001 |
| **Obesity-related cancer** | 0.112 | 0.047 | 0.079 |
| **Broad definition of alcohol-related cancer** | 0.053 | <0.001 | 0.003 |
| **Total cancer** | 0.028 | 0.016 | 0.007 |
| *Previous drinkers were excluded from the analysis.  Model is adjusted for baseline age and sex.  Body Fat (BF%) was measured by bioimpedance using the Tanita BC-418MA device (Tanita, Tokyo, Japan). Waist circumference (WC) was measured by using flexible plastic tape with the participant in the resting-standing position by a trained professional. Body mass index (BMI) = Weight (kg)/height (m^2^).  Smoking status was categorized as never, previous, and current smokers. | | | |

**Supplementary Table 12.** Joint association of smoking status and alcohol consumption with alcohol-related cancer incidence

|  | **Narrow definition of alcohol-related cancer**  HR (95% CI) | **Broad definition of alcohol-related cancer**  HR (95% CI) |
| --- | --- | --- |
| **Never smoker** |  |  |
| Never drinker | - | - |
| Previous drinker | 1.13 (0.98, 1.30) | 1.11 (0.97, 1.27) |
| Within guideline | 1.06 (0.97, 1.15) | 1.07 (0.99, 1.16) |
| Above guideline | 1.07 (0.97, 1.17) | 1.06 (0.97, 1.15) |
| **Previous smoker** |  |  |
| Never drinker | 0.97 (0.78, 1.20) | *1.21 (1.01, 1.45)* |
| Previous drinker | *1.34 (1.17, 1.54)* | *1.58 (1.40, 1.78)* |
| Within guideline | *1.20 (1.10, 1.31)* | *1.33 (1.23, 1.45)* |
| Above guideline | *1.34 (1.22, 1.46)* | *1.47 (1.35, 1.60)* |
| **Current smoker** |  |  |
| Never drinker | 1.21 (0.91, 1.61) | *2.06 (1.68, 2.53)* |
| Previous drinker | *1.55 (1.28, 1.88)* | *2.56 (2.22, 2.96)* |
| Within guideline | *1.20 (1.08, 1.33)* | *1.86 (1.70, 2.03)* |
| Above guideline | *1.55 (1.40, 1.72)* | *2.18 (1.99, 2.38)* |
| Cox proportional hazard model. Never drinker I Never smoker is the referent group.  Model is adjusted for baseline age, sex, dietary pattern score (determined by higher consumption of fruit, vegetables, and fish and lower consumption of processed meats and red meats (Rutten-Jacobs et al., 2018)), sleep duration (hrs/night), education, BMI, Townsend Deprivation Index and physical activity ((MET)-hour/week), chronic diseases (major cardiovascular disease (ICD-10 codes I00 to I99), Type 2 diabetes (ICD-10 codes E11.0 to E11.9 and E12) and dyslipidaemia (ICD-10 codes E78.0-E78.6) diagnosed by a doctor and hospital admission records and self-reported cardiovascular diseases and Type 2 diabetes).  Alcohol consumption categories are based on the average weekly intake of standard drinks relative to UK guidelines. In the UK, one standard drink equals to 8 g of pure alcohol. Within guidelines: ≤ 14 units/week; above guidelines:>14 units/week.  Alcohol-related cancer according to the broad definition included oral cavity, throat, larynx, esophagus, liver, colorectal, stomach, female breast, pancreas and lung cancer (IARC, 2014).  Alcohol-related cancer according to the narrow definition included oral cavity, throat, larynx, esophagus, liver, colorectal, stomach, female breast (IARC, 2014).  *Italic font indicates statistical significance (p<0.05).* | | |

**Appendix.** Cancer diagnosis codes: based on the International Classification of Diseases (ICD), 9th Revision (ICD-9) and 10th Revision (ICD-10) diagnosis code

|  | **ICD-9** | **ICD-10** |
| --- | --- | --- |
| **Broad definition of alcohol-related cancer** | | |
| Oral cavity | 141-145 | C00 – C08 |
| Throat | 146-149 | C09 – C14 |
| Larynx | 161 | C32 |
| Esophagus | 150 | C15 |
| Liver | 155 | C22 |
| Colorectal | 152 – 154 | C18 – C20 |
| Stomach | 151 | C16 |
| Female Breast | 174 | C50 |
| Pancreas | 157 | C25 |
| Lung | 162 | C34 |
| **Narrow definition of alcohol-related cancer** | | |
| Oral cavity | 141-145 | C00 – C08 |
| Throat | 146-149 | C09 – C14 |
| Larynx | 161 | C32 |
| Esophagus | 150 | C15 |
| Liver | 155 | C22 |
| Colorectal | 152 – 154 | C18 – C20 |
| Stomach | 151 | C16 |
| Female Breast | 174 | C50 |
| **Obesity-related cancer** | | |
| Esophagus | 150 | C15 |
| Gastric cardia | 151.0 | C16.0 |
| Colorectal | 152-154 | C18 – C20 |
| Liver | 155 | C22 |
| Gallbladder | 156 | C23 |
| Pancreas | 157 | C25 |
| Female Breast | 174 | C50 |
| Corpus uteri | 182 | C54 |
| Ovary | 183 | C56 |
| Kidney: renal-cell | 189 | C64 |
| Meningioma | 192.1 – 192.3 | C70 |
| Thyroid | 193 | C73 |
| Multiple myeloma | 203 | C90.0 |
| **Total cancer** | 140.0-194.9/ 199.0-209.3 | C0-C6, C70 - C75, C7A, C8-C9 |
